# Selective and Sequential Catalytic Chemical Depolymerization and Upcycling of Mixed Plastics

**DOI:** 10.1021/acsmacrolett.3c00751

**Published:** 2024-01-22

**Authors:** Adam J. Spicer, Arianna Brandolese, Andrew P. Dove

**Affiliations:** School of Chemistry, University of Birmingham, Edgbaston, Birmingham B15 2TT, United Kingdom

## Abstract

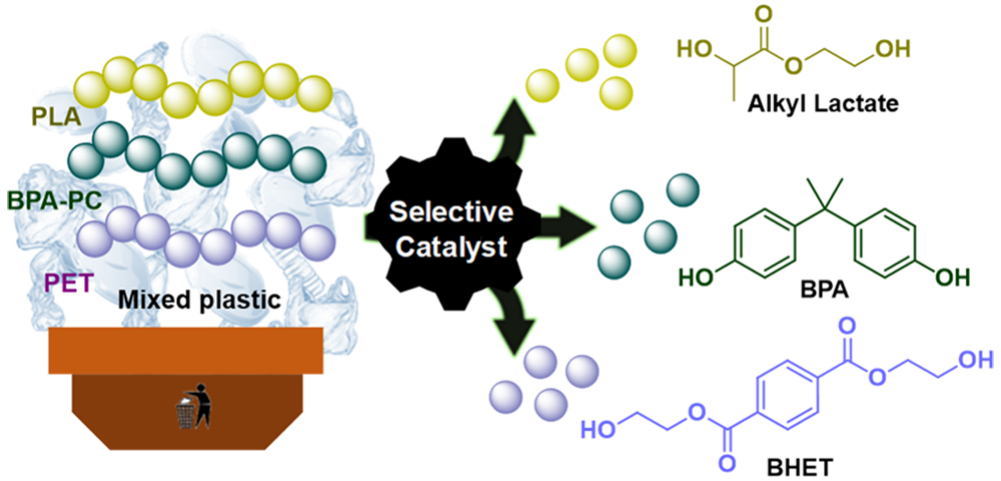

Chemical recycling to monomer (CRM) provides a useful technique to allow for polymer-to-monomer-to-polymer circular economies. A significant challenge remains, however, in the treatment of mixed plastics by CRM in which unselective depolymerization requires either presorting of plastics or purification processes postdepolymerization, both of which add cost to waste plastic processing. We report a simple, yet selective, chemical depolymerization of three commonly used polymers, poly(lactic acid) (PLA), bisphenol A polycarbonate (BPA-PC), and polyethylene terephthalate (PET), using inexpensive and readily available common metal salt/organobase dual catalysts. By a judicious choice of catalyst and conditions, selective and sequential depolymerization of mixtures of the polymers was demonstrated. Furthermore, the potential for upcycling of polymers to value-added monomers was explored through the application of alternative nucleophiles within the depolymerization.

As our consumption of plastic continues to grow rapidly, so does the generation of plastic waste. It has been estimated that by 2060 between 155 and 265 million tons of commodity plastic waste will be generated annually across the globe.^1^ With vast quantities of plastic waste being produced, methods to capture and recycle them are not efficient, and hence the majority of plastic waste that is generated goes to landfills or is incinerated. Not only does this lead to environmental pollution but also the linear economy represents a loss of inherent material value. Therefore, solutions that can prevent waste generation and circularize the material value of plastics are highly sought after.

Currently, postconsumer plastic waste (PCPW) that is recycled is primarily processed by mechanical recycling methods. These are widely implemented, easy to access, and cheap to run;^2^ however, they are inefficient^3^ and are limited by thermal degradation of the polymer chains.^4^ Mechanical recycling processes are also dependent on the presorting of PCPW streams to reduce contamination and maintain recyclate quality. However, perfect sorting is not possible as many commonly used plastics (such as those with carbonyl containing backbones) are often hard to distinguish by both manual methods and automated infrared sorting.^5^ Furthermore, plastics that are intractably mixed or are contaminated by additives such as dyes or plasticizers lower the quality of the recyclate, leading to downcycling, i.e., creating lower value plastic.^6−8^

Chemical recycling to monomer (CRM) offers an appealing part of the solution. Through CRM, colored waste plastics can be returned to virgin-quality plastic after the separation of monomers and additives.^9^ Furthermore, the potential to leverage the diverse reactivity of different functional groups within polymer backbones presents an opportunity to deliver selective CRM, which could provide benefits to global recycling efforts by reducing the need for labor-intensive manual sorting of plastic wastes.

Selective CRM does not require any presorting steps, as separation is achieved by exploiting chemical reactivity differences between each constituent plastic within the mixture to be processed. Therefore, plastic impurities (such as PLA or BPA-PC) can be removed from the waste stream of a desired plastic (such as PET) while also undergoing upcycling in the process. In comparison to the chemical recycling to monomers of single plastic systems, selective CRM is less prominent in the literature. So far, only limited examples of selective CRM have been reported, involving the methanolysis,^10,11^ hydrosilylation,^12−14^ hydrogenolysis,^15^ and most recently both glycolysis and alcoholysis processes^16−20^ of up to two mixed polymeric formulations. To date, only one example has been recently reported on the selective deconstruction of four mixed plastics by an organocatalyst in which different temperatures were leveraged to enable the selectivity observed.^21^

Herein, we extend the concept of selective CRM to a ternary mixture of three commonly used carbonyl-containing plastics, PLA, BPA-PC, and PET, that are all susceptible to hydrolytic/alcoholytic degradation (Figure 1). Sequential and selective catalytic glycolysis of mixtures of all three plastics was achieved using simple and inexpensive single- and dual-catalyst systems consisting of metal salts and organic bases. In our developed protocol, catalytic systems do not require any prior catalyst synthesis or catalyst pretreatment, thus simplifying the depolymerization procedure.

**Figure 1:**
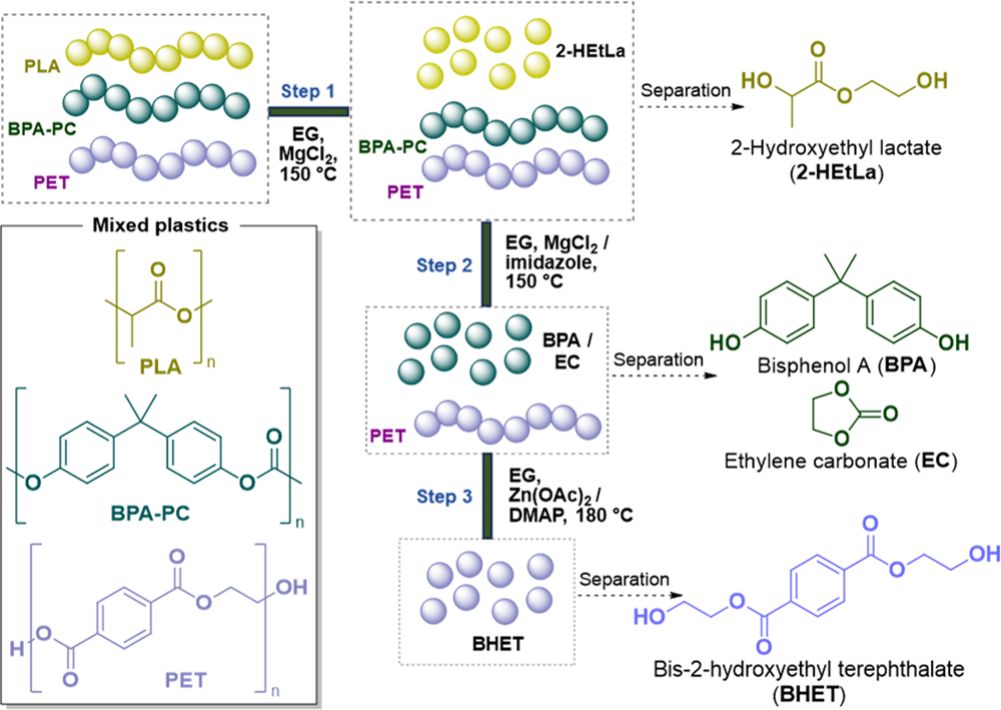
Overview of the three-step selective and sequential depolymerization process that was designed using ethylene glycol (EG) between PLA, BPA-PC, and PET.

Furthermore, we have shown that the application of alternate nucleophiles allows for the selective upcycling of single plastics within these mixtures.

In order to identify catalysts and conditions that could be implemented in a multistep selective depolymerization process, a simple screen was developed to assess the depolymerization kinetics of PLA, BPA-PC, and PET individually. This was achieved by glycolysis using excess ethylene glycol (EG), at three discrete temperatures (120, 150, and 180 °C), with a range of single- and dual-catalyst systems under each condition. Depolymerization kinetics were monitored by ^1^H NMR spectroscopy over a 2 h period, integrating the peak corresponding to the reference proton environment within each product against *N*-methylpyrrolidinone (NMP, δ = 2.71 ppm) used as an internal standard (see Figures S17, S18, and S19). PET conversion to bis-2-hydroxyethyl terephthalate (BHET) was monitored using the aromatic singlet peak at δ = 8.10 ppm; PLA conversion to 2-hydroxyethyl lactate (2-HetLa) was determined from the methyl doublet peak at δ = 1.24 ppm, and BPA-PC conversion to bisphenol A (BPA) was calculated from the average integration of the two doublet peaks which arise at δ = 6.95 and 6.65 ppm, respectively. Notably, when analyzing the depolymerization kinetics of BPA-PC, a side product was commonly observed, which, in accordance with previous literature,^16^ is assigned as bisphenol A bis(2-hydroxyethyl) ether (BPA-SP). BPA-SP formation can negatively affect the conversion of BPA-PC to the target monomer BPA; therefore, total BPA-PC conversion was taken as the sum of average conversion to BPA and average conversion to BPA-SP.

A benefit of using a dual-catalyst system that includes a metal-based Lewis acid component arose from the possibility of tuning different properties including Lewis acidity of the metal (*e.g.*, Mg *vs* Zn), size of the metal, and metal ligands/counteranions (*e.g.*, acetate *vs* chloride) that in turn will provide a handle to moderate activity.^22,23^ An initial screen of catalysts was therefore undertaken with the dual-catalyst systems comprising a Lewis acid selected from Zn(OAc)_2_, Mg(OAc)_2_, MgCl_2_, and either 4-dimethylamino pyridine (DMAP) or imidazole (Imid) as base (Tables 1 and 2), chosen on account of their ability to work as synergistic catalysts.^19^ The synergistic effect was exploited by the simultaneous presence of the Lewis acids, involved in the activation of the electrophile (through the binding to the carbonyl group, thus facilitating the attack of the ethylene glycol),^24^ and the bases, involved in the activation of the nucleophile. Prior to exploring the activity of the dual-catalyst systems toward the glycolysis of the three plastics, each individual Lewis acid and base component was screened as a control (Table 1). Depolymerizations were explored using 15 mol % of catalyst to compare catalysts’ efficiency with previously reported studies.^18^ Of the five single-catalyst systems investigated, all three Lewis acids showed good ability to mediate the depolymerization of PET, PLA, and BPA-PC, especially at 180 °C. At lower temperature, the catalysts’ activity typically decreased, although the reduction in activity was less pronounced for PLA.

**Table 1:** Complete Data for the Screening of PET, PLA, and BPA-PC Depolymerization Kinetics at 180, 150, and 120 °C Using Ethylene Glycol and Single-Catalyst Systems

			Final conversion (%)^a^			BPA:SP (%)^c^	
Entry	Catalyst	*T* (°C)	PET	PLA	BPA-PC^b^	BPA	SP
1	Zn(OAc)_2_	180	83	97	95	86	9
2	Zn(OAc)_2_	150	0	99	92	90	2
3	Zn(OAc)_2_	120	0	22	0	0	0
4	Mg(OAc)_2_	180	62	97	94	87	7
5	Mg(OAc)_2_	150	5	85	83	79	4
6	Mg(OAc)_2_	120	0	13	1	1	0
7	MgCl_2_	180	18	99	98	91	7
8	MgCl_2_	150	0	97	0	0	0
9	MgCl_2_	120	0	0	0	0	0
10	DMAP	180	94	88	89	47	42
11	DMAP	150	30	99.6	98	70	28
12	DMAP	120	0	99.1	67	56	11
13	Imid	180	17	94	96	66	30
14	Imid	150	1	90	68	59	9
15	Imid	120	0	0	0	0	0

a Final conversion of polymer to monomer after *t* = 2 h using 15 mol % of catalyst.

b Total conversion of BPA-PC.

c Percentage ratio of conversion of BPA-PC to BPA or BPA-SP.

**Table 2:** Complete Data for the Screening of PET, PLA, and BPA-PC Depolymerization Kinetics at 180, 150, and 120 °C Using Ethylene Glycol Dual-Catalyst Systems

			Final conversion (%)^a^			BPA:SP (%)^c^	
Entry	Catalyst	*T* (°C)	PET	PLA	BPA-PC^b^	BPA	SP
1	Zn(OAc)_2_/DMAP	180	98	96	98	62	36
2	Zn(OAc)_2_/DMAP	150	36	96	97	74	23
3	Zn(OAc)_2_/DMAP	120	1	73	54	54	0
4	Mg(OAc)_2_/DMAP	180	66	92	97	77	20
5	Mg(OAc)_2_/DMAP	150	20	88	97	87	10
6	Mg(OAc)_2_/DMAP	120	0	55	37	36	1
7	MgCl_2_/DMAP	180	97	97	94	81	13
8	MgCl_2_/DMAP	150	18	99	93	82	11
9	MgCl_2_/DMAP	120	0	12	58	57	1
10	Zn(OAc)_2_/Imid	180	82	91	94	66	28
11	Zn(OAc)_2_/Imid	150	9	94	95	82	13
12	Zn(OAc)_2_/Imid	120	0	33	2	2	0
13	Mg(OAc)_2_/Imid	180	71	94	95	80	15
14	Mg(OAc)_2_/Imid	150	6	86	88	82	6
15	Mg(OAc)_2_/Imid	120	0	13	6	6	0
16	MgCl_2_/Imid	180	69	99.5	96	85	11
17	MgCl_2_/Imid	150	3	99	99	95	4
18	MgCl_2_/Imid	120	0	8	8	8	0

a Final conversion of polymer to monomer after *t* = 2 h using 15 mol % of catalyst.

b Total conversion of BPA-PC.

c Percentage ratio of conversion of BPA-PC to BPA or BPA-SP.

The Lewis bases displayed similar initial results, with higher conversions at 180 °C, while at lower temperature, the glycolysis was accompanied by lower polymer conversion (Table 1, entries 10–15). Nevertheless, DMAP proved to be more active than imidazole for the depolymerization, especially when employed for PET at 180 °C (Table 1, entry 10), promoting 94% PET conversion to BHET, whereas the imidazole-catalyzed depolymerization (Table 1, entry 13) reached only 17% conversion within 2 h. Furthermore, the complete glycolysis of PLA to 2-HEtLa in the presence of DMAP was observed in 2 h at 120 °C (Table 1, entry 12). In contrast, BPA-PC glycolysis at 180 °C conducted in the presence of imidazole led to a higher polymer depolymerization conversion than that in the presence of DMAP (96% *vs* 89%; Table 1, entries 10 and 13) along with a higher ratio of BPA over BPA-SP formation (66% BPA *vs* 47% BPA). Overall, the single-catalyst screening furnished information about the selectivity of each catalytic system for a single polymeric formulation. Surprisingly, MgCl_2_ (Table 1, entry 8) was able to promote the complete depolymerization of PLA at 150 °C, while both BPA-PC and PET were left unreacted under the same reaction conditions. Further kinetic studies based on the use of dual-catalyst systems were thus exploited with the aim of selectively depolymerizing BPA-PC and PET (Table 2).

As observed, when a single catalyst was used, almost full depolymerization of PLA was obtained at 150 and 180 °C when both Lewis acids and bases are present. In the case of PET, dual-catalyst systems including the Lewis base, DMAP, were more active compared to their imidazole counterparts, with full PET conversion (98%) being achieved by our benchmark catalyst system,^19^ Zn(OAc)_2_/DMAP, at 180 °C (Table 2, entry 1). Although in a fashion similar to that observed with a single catalyst, PET conversion to BHET was highly influenced by working temperature, with lower reaction temperatures greatly affecting final conversion levels. BPA-PC depolymerization was also nearly quantitative in all cases at 150 °C and above, while the ratio of BPA to BPA-SP formation varied throughout the different reaction conditions. The highest BPA-PC glycolysis conversion after a 2 h reaction time was achieved using MgCl_2_ paired with imidazole at 150 °C (Table 2, entry 17), which also coincidentally displayed both the lowest BPA-SP formation levels of any dual-catalyst systems that facilitated complete conversion of BPA-PC. More importantly, this catalyst combination displayed near complete inactivity for PET depolymerization. Ultimately, the screening study highlighted three candidate reaction systems that could be employed in a selective, sequential depolymerization process. These were identified as (1) MgCl_2_ at 150 °C, for the selective depolymerization of PLA, followed by (2) MgCl_2_/imidazole at 150 °C to depolymerize BPA-PC, and finally (3) Zn(OAc)_2_/DMAP at 180 °C to depolymerize the residual PET.

Prior to performing a selective sequential depolymerization process, each step was individually evaluated, assessing any issues resulting from the simultaneous presence of more than one polymeric formulation in the reaction mixture. Indeed, in the first step, the contemporaneous presence of both BPA-PC and PET alongside PLA affected the ability of the PLA pellets to solubilize, therefore slowing the rate of the depolymerization process. Nevertheless, increasing the reaction time to 4 h helped to achieve the full glycolysis of PLA. Similarly, the reaction time was extended for BPA-PC glycolysis to 4 h, while PET depolymerization was complete within 2 h.

Lastly, with the aim of conducting a continuous process, all three steps were performed in sequence; thus, polymer pellets used in step 1 were subsequently carried through each step, with washing (to remove the soluble depolymerization products and catalysts) and drying of unreacted pellets in between each depolymerization step. Using the optimized reaction conditions for step 1, 99% conversion of PLA to 2-HEtLa was achieved in 4 h, with no evidence of PET or BPA-PC glycolysis (Figure 2A). Unreacted BPA-PC and PET pellets were thus removed from the reaction mixture, washed with EG, and dried, before immediately being transferred to a reaction vessel containing the MgCl_2_/imidazole catalyst system used in step 2, which had been solubilized in EG and preheated to the working temperature. After 4 h, almost quantitative conversion (93%) of BPA-PC was detected with only marginal PET conversion (6%), likely a result of the prolonged reaction time (Figure 2B). Residual PET was last removed from the reaction mixture and washed with EG and added to a preheated reaction mixture of the Zn(OAc)_2_/DMAP dual-catalyst system in EG, achieving in 2 h almost quantitative conversion (98%) of PET to BHET (Figure 2C). Importantly, this methodology allows for separation of the alcoholysis products from residual polymers by simple filtration with no requirement for the separation of complex mixtures of depolymerization products.

**Figure 2:**
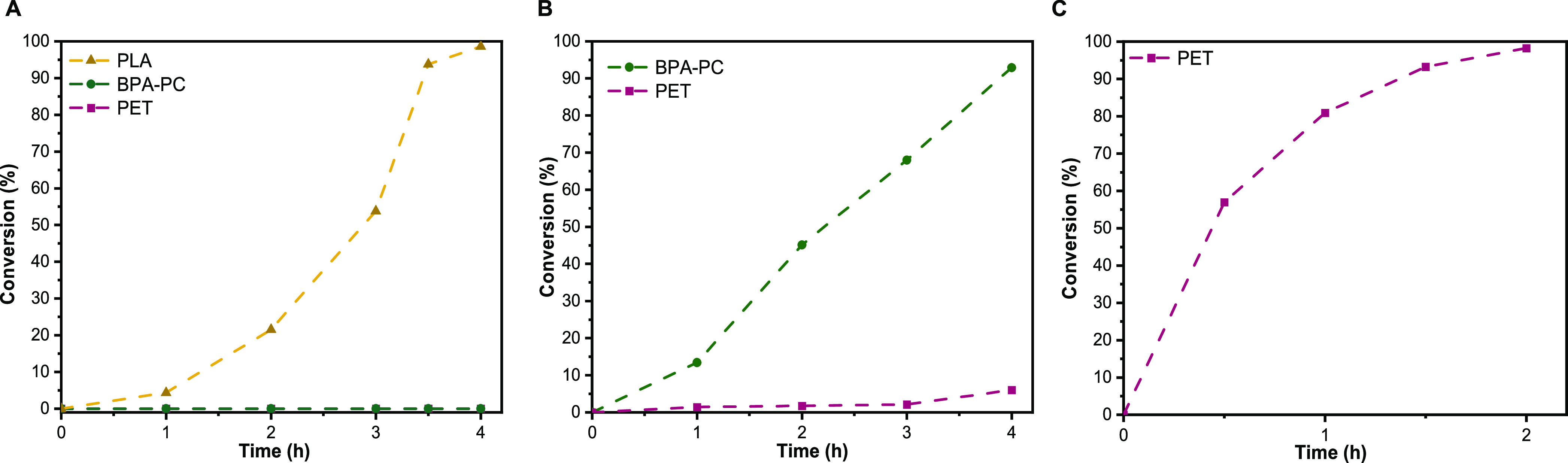
Conversion/time plots for (A) selective PLA depolymerization at 150 °C using MgCl_2_; (B) selective BPA-PC depolymerization at 150 °C using the MgCl_2_/imidazole catalytic system; and (C) PET depolymerization at 180 °C using the Zn(OAc)_2_/DMAP catalytic system.

Following the same proof-of-concept selective depolymerization methodology, we sought to explore the use of alternative nucleophiles to EG in both PLA and BPA-PC depolymerization to access potentially value-added monomers (Table 3).

**Table 3:** Complete Data for the Screening of Alternative Nucleophiles to EG for the Depolymerization of PLA and BPA-PC in Steps 1 and 2

Entry	Nu.^a^	Cat.^b^	*T* (°C)	*t* (h)	PLA (%)^c^	BPA-PC (%)^c^	PET (%)^c^
1	EG	MgCl_2_	150	4	99	0	0
2	EG	MgCl_2_/Imid	150	4	N/A	93	6
3	TMPAE	MgCl_2_/Imid	150	2	N/A	99	0
4	Geraniol	MgCl_2_	150	6	96	15	0
5	Nerol	MgCl_2_	150	6	68	23	1
6	1-Hexanol	MgCl_2_	150	1	99	6	0
7	1-Octanol	MgCl_2_	150	5	99	78	0

a Nucleophile.

b Catalyst system.

c Total average conversion.

Several different nucleophiles, including primary alcohols, longer-chain diols, and naturally occurring alcoholic monoterpenoids were employed. Using optimized catalytic conditions, the selective upcycling of BPA-PC over PET was achieved with the use of an allyl-functionalized diol, trimethylolpropane allyl ether diol (TMPAE), that has previously been used for the preparation of allyl-functionalized cyclic carbonates.^16,25^ Herein, TMPAE was shown to quantitatively selectively depolymerize BPA-PC over PET (Table 3, entry 3), in the presence of MgCl_2_/imidazole (2 h reaction time).

The obtained cyclic carbonate 2-allyloxymethyl-2-ethyltrimethylene carbonate (AOMEC) (Figure 3A) represents a valuable monomer that can undergo ring-opening polymerization (ROP) to prepare polycarbonates containing an additional functional group in the repeating unit amenable to further derivatization (e.g., through thiol–ene click chemistry).

**Figure 3:**
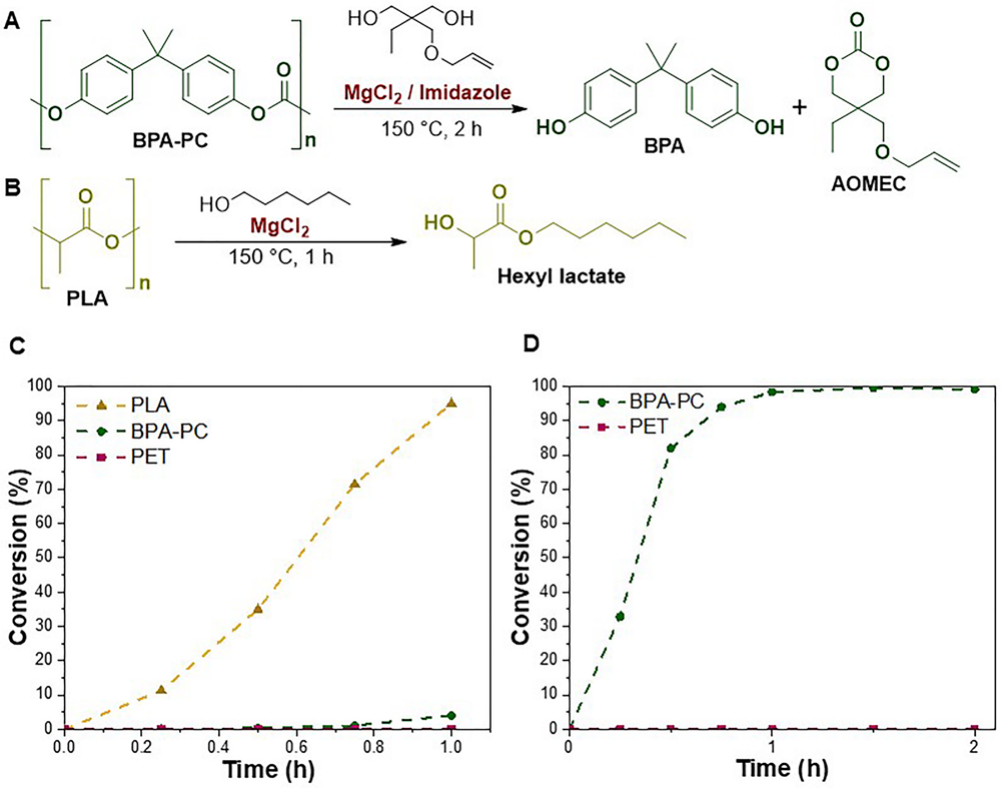
(A) Formation of hexyl lactate from PLA using 1-hexanol. (B) Formation of AOMEC from BPA-PC using TMPAE. Conversion/time plots for the selective alcoholysis and upcycling of PLA over BPA-PC and PET using 1-hexanol at 150 °C in the presence of MgCl_2_ (C) and BPA-PC over PET using TMPAE at 150 °C in the presence of MgCl_2_/imidazole (D).

Depolymerization of PLA in the presence of geraniol and nerol terpenes under the optimized catalyst conditions resulted in the alcoholysis of PLA over BPA-PC being preferred, although some conversion of BPA-PC to the corresponding terpene-based carbonate was observed. Again, PET remained untouched under these conditions (Table 3, entries 4 and 5).

We hypothesized that the apolar nature of the selected terpene-based alcohols enabled greater solubility of the BPA-PC in the reaction medium and hence sought to study more polar solvents. To that end, we studied 1-hexanol (Figure 3B). Quantitative PLA depolymerization (>99%) was complete within 1 h (Table 3, entry 6), while both BPA-PC and PET were almost unreacted (only 6% BPA formation). Interestingly, the hexyl lactate product is a natural flavoring molecule found in wine, which can also be employed as an additive in the food industry.^26,27^ Furthermore, the latter can be used as a starting material in the formation of higher poly(alkyl lactate acrylate)s, acting as viscosity index modifiers in bioderived lubricating oils.^28,29^ Decreasing the polarity of the reaction medium by using 1-octanol in the presence of a MgCl_2_ single-catalyst system promoted the simultaneous full depolymerization of BPA-PC with a good yield (78%) as anticipated, demonstrating the importance of the reactant choice for achieving selectivity (Table 3, entry 7).

Lastly, a continuous selective depolymerization and upcycling process was conducted by using 1-hexanol and TMPAE as nucleophiles. While 1-hexanol was used in the first step in the presence of MgCl_2_, TMPAE was instead used in the second step in the presence of a MgCl_2_/imidazole dual-catalyst system. The first step achieved complete (99%) conversion of PLA to hexyl lactate, with no PET degradation observed and only minimal BPA formation detected (4%) (Figure 3C). Likewise, step 2 successfully demonstrated the complete (99%) conversion of BPA-PC pellets carried over from step 1 to BPA and AOMEC entirely selectively over PET, which remained unreacted throughout (Figure 3D). The third step was last performed as above through the quantitative depolymerization of PET (99%) to BHET within 2 h.

Using a simple binary dual-catalysis approach, the three-step selective and sequential chemical depolymerization of ternary mixtures of common plastics, PLA, BPA-PC, and PET has been achieved. This proof of principle demonstrates that a catalytic chemical depolymerization cascade can be applied to mixed heteroatom-containing plastics with similar, albeit distinct, chemical reactivity. These approaches could be important to help achieve a plastics circular economy by either enabling the purification of contaminated high value waste streams, such as PET, or addressing some of the most challenging plastic wastes such as multilayer films and composite materials, such as those found in the automotive industry. Selectivity was achieved here by using inexpensive and commercially available common metal salt/organobase dual catalysts, and through choice of the suitable catalyst and reaction conditions, a fast selective and sequential chemical recycling was exploited with quantitative conversions in all the steps. We also demonstrated selective upcycling from these mixed polymers, which shows that such complex plastic wastes could constitute an alternative resource for the synthesis of new added-value molecules and monomers.

## Supporting Information Available

Experimental methods, synthetic procedures and spectroscopic data (^1^H NMR), and kinetic study (PDF)
