# Supplementary material for: Selective and Sequential Catalytic Chemical Depolymerization and Upcycling of Mixed Plastics
Source: ACS Macro Lett. 2024 Jan 22;13(2):189–94. doi: 10.1021/acsmacrolett.3c00751 (PMC10883033; doi:10.1021/acsmacrolett.3c00751)
Supplement: Supplementary file 1 — mz3c00751_si_001.pdf [file mz3c00751_si_001.pdf]

Supporting Information

Selective and Sequential Catalytic Chemical Depolymerisation and Upcycling of Mixed Plastics

Adam. J. Spicer, Arianna Brandolese and Andrew. P. Dove*

Mr A. J. Spicer, Dr A. Brandolese and Prof. A. P. Dove
School of Chemistry
University of Birmingham
Edgbaston, Birmingham, B15 2TT, United Kingdom
E-mail: a.dove@bham.ac.uk

List of abbreviations

2-HetLa	2-hydroxyethyl lactate
AEOMEC	2-allyloxymethyl-2-ethyltrimethylene carbonate
BHET	Bis-2-hydroxyethyl terephthalate
BPA	Bisphenol A
BPA-PC	Bisphenol A-based polycarbonate
BPA-SP	Bisphenol A bis(2-hydroxyethyl) ether
DMAP	4-dimethylamino pyridine
DMSO	Dimethyl sulfoxide
EG	Ethylene glycol
NMP	N-methyl pyrrolidinone
NMR	Nuclear magnetic resonance
PET	Poly(ethylene terephthalate)
PLA	Polylactic acid
TMPAE	Trimethylolpropane allyl ether diol

Table of Content

General information	S4
General procedure for the kinetic study of the selective glycolysis	S5
Kinetic studies of PLA, BPA-PC and PET depolymerization using single catalyst system	S5
Kinetic studies of PLA, BPA-PC and PET depolymerization using dual catalyst system	S10
Kinetic studies for the selective PLA, BPA-PC and PET depolymerization using single and dual catalyst systems	S20
Sequential selective depolymerisation reactions in presence of ethylene glycol	S21
Kinetic studies for selective depolymerization with alternative nucleophiles	S24
Sequential selective depolymerisation reactions in presence of alternative nucleophiles	S25
Comparisons of Time/ Conversion plots for each step of the three selective depolymerisation routes	S26
References	S28

General information

All chemicals were commercially available (purchased from Sigma-Aldrich) and used without further purification (unless otherwise stated). PET, PLA and BPA-PC were purchased from Goodfellow Cambridge Limited and used in the form of white pellets in all experiments (pellets size: 3 − 5 mm). 1-Methyl-2-pyrrolidinone was purified by distillation under vacuum using a Schlenk line.

All NMR spectroscopy experiments were performed at 25 °C on a Bruker DPX-300/400 NMR instrument operating at 300 or 400 MHz for ^{1}H (100.57 MHz for ^{13}C). ^{1}H NMR spectra are referenced to residual proton solvent (δ_H = 2.50 for DMSO-d_6 and δ_H = 7.26 for $CDCl_3$). The resonance multiplicities are described as s (singlet), d (doublet), dd (doublet of doublets), t (triplet), q (quartet) or m (multiplet).

Flash column chromatography was carried out on silica gel purchased from Merck (High purity grade, 0.035 – 0.070 mm, 60 Å), using reagent grade solvent purchased from Sharlau or Fisher Scientific.

Experimental Procedures

General procedure for the kinetic study of the selective glycolysis

A general procedure was followed for all glycolysis reactions and all reactions were completed in triplicate, to allow for conversion levels to be averaged. Reactions were carried out in 20 mL scintillation vials equipped with magnetic stirrers, each vial was charged with the catalyst(s) (0.375 mmol, 15 mol% of each), ethylene glycol (50 mmol, 2000 mol%) and the internal standard NMP (0.025 mmol, 10 mol%). The vials were sealed and placed within a heating block, pre-heated to the desired temperature. The depolymerisation reactions were stirred at 500 rpm, for 15 minutes to solubilise the catalysts and allow the solvent to reach temperature. After 15 minutes, an aliquot (0.2 mL) was removed from the reaction mixture to act as a t_0 for ^{1}H NMR spectroscopic analysis, pelletised polymer (2.5 mmol) was then added, and the vials were resealed. Further aliquots for ^{1}H NMR spectroscopy were taken at t = 0.25, 0.5, 0.75, 1, 1.5 and 2 h and dissolved in DMSO-d_6. At t = 2 h, the vials were removed from the heating block, the magnetic stirrer was removed, and the sealed vials were placed in the fridge overnight to cool down pending further work up.

Kinetic studies of PLA, BPA-PC and PET depolymerization using single catalyst systems

$Zn(OAc)_2{\cdot}2H_2O$

Following the general procedure, the kinetics of the depolymerization of PLA, BPA-PC and PET in EG using 0.15 equiv. of $Zn(OAc)_2$ single catalyst system was conducted at 120 °C, 150 °C and 180 °C. Total BPA-PC conversion was taken as the sum of conversion to BPA and conversion to BPA-SP.

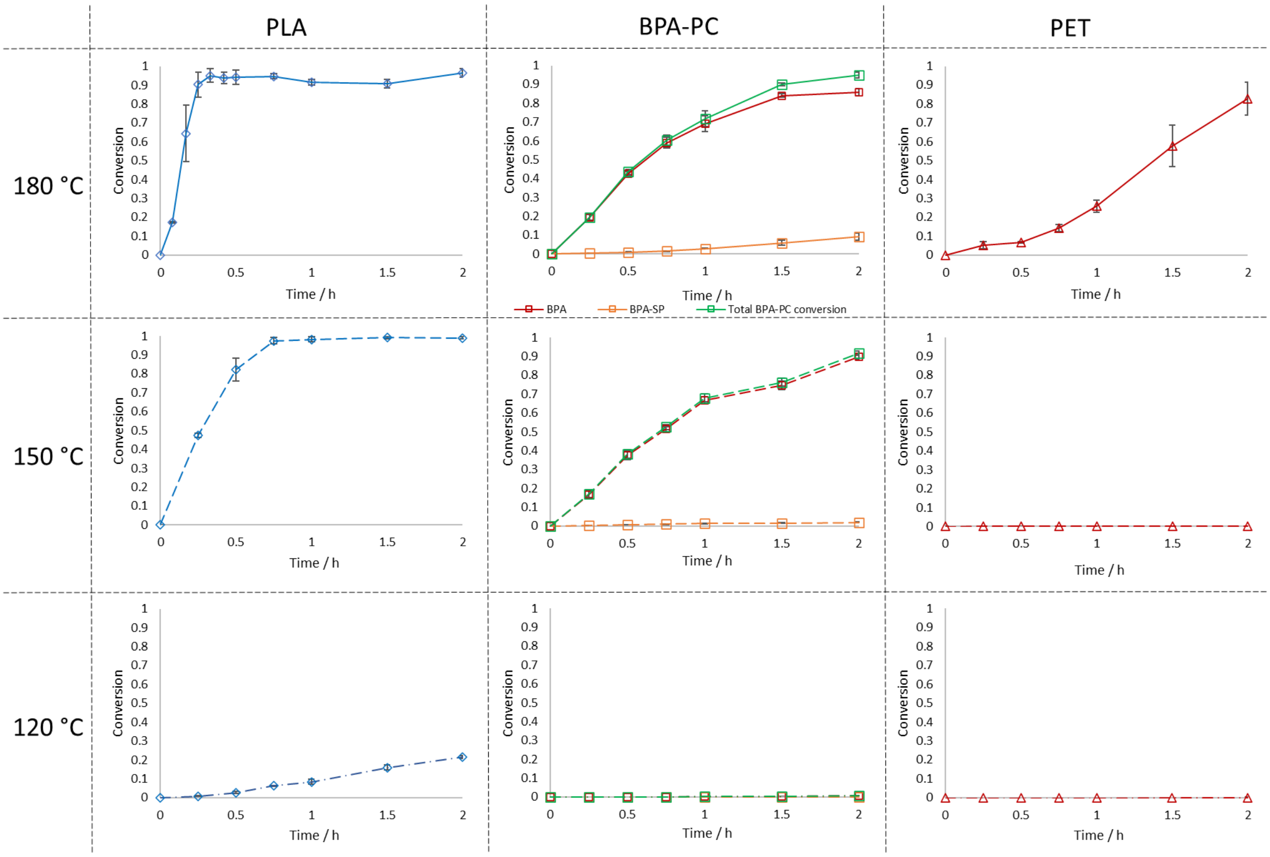

Figure S1. Kinetic plots of conversion against time for depolymerisation of PLA, BPA-PC and PET at 120 °C, 150 °C and 180 °C using zinc acetate as single catalyst system. The kinetics were followed by ^{1}H NMR spectroscopy in DMSO-d_6. Conversions of PLA to 2-HEtLa (δ_H = 1.24 (d, 3H)), BPA-PC to BPA (δ_H = 6.90 (d, 4H), 6.65 (d, 4H)) and PET to BHET (δ_H = 8.10 (s, 4H)) were determined using NMP as an internal standard (δ_H = 2.71 (s, 3H)).

$Mg(OAc)_2 \cdot 4H_2O$

Following the general procedure, the kinetics of the depolymerization of PLA, BPA-PC and PET in EG using 0.15 equiv. of $Mg(OAc)_2$ single catalyst system was conducted at 120 °C, 150 °C and 180 °C. Total BPA-PC conversion was taken as the sum of conversion to BPA and conversion to BPA-SP.

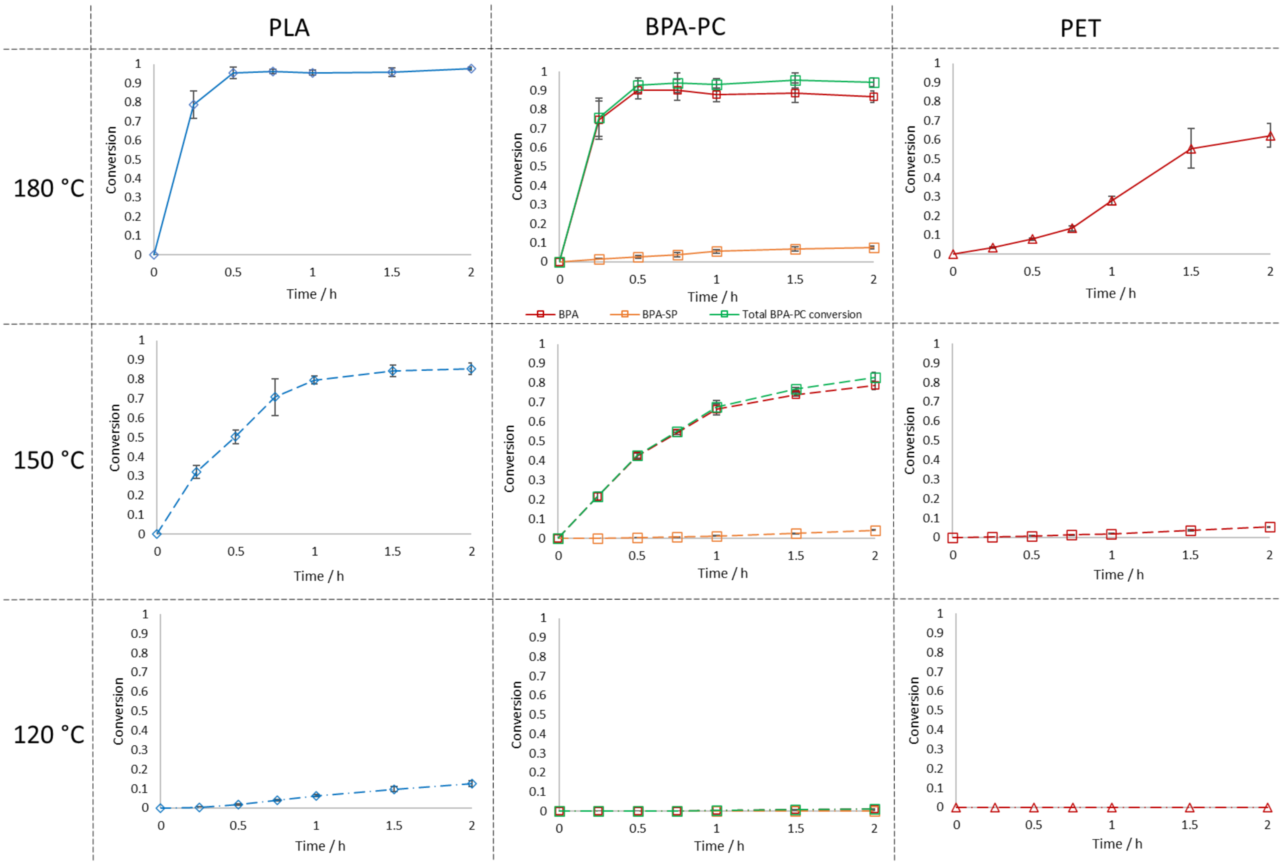

Figure S2. Kinetic plots of conversion against time for depolymerisation of PLA, BPA-PC and PET at 120 °C, 150 °C and 180 °C using magnesium acetate as single catalyst system. The kinetics were followed by ^{1}H NMR spectroscopy in DMSO-d_6. Conversions of PLA to 2-HEtLa (δ_H = 1.24 (d, 3H)), BPA-PC to BPA (δ_H = 6.90 (d, 4H), 6.65 (d, 4H)) and PET to BHET (δ_H = 8.10 (s, 4H)) were determined using NMP as an internal standard (δ_H = 2.71 (s, 3H)).

MgCl$_2$

Following the general procedure, the kinetics of the depolymerization of PLA, BPA-PC and PET in EG using 0.15 equiv. of MgCl$_2$ single catalyst system was conducted at 120 °C, 150 °C and 180 °C. Total BPA-PC conversion was taken as the sum of conversion to BPA and conversion to BPA-SP.

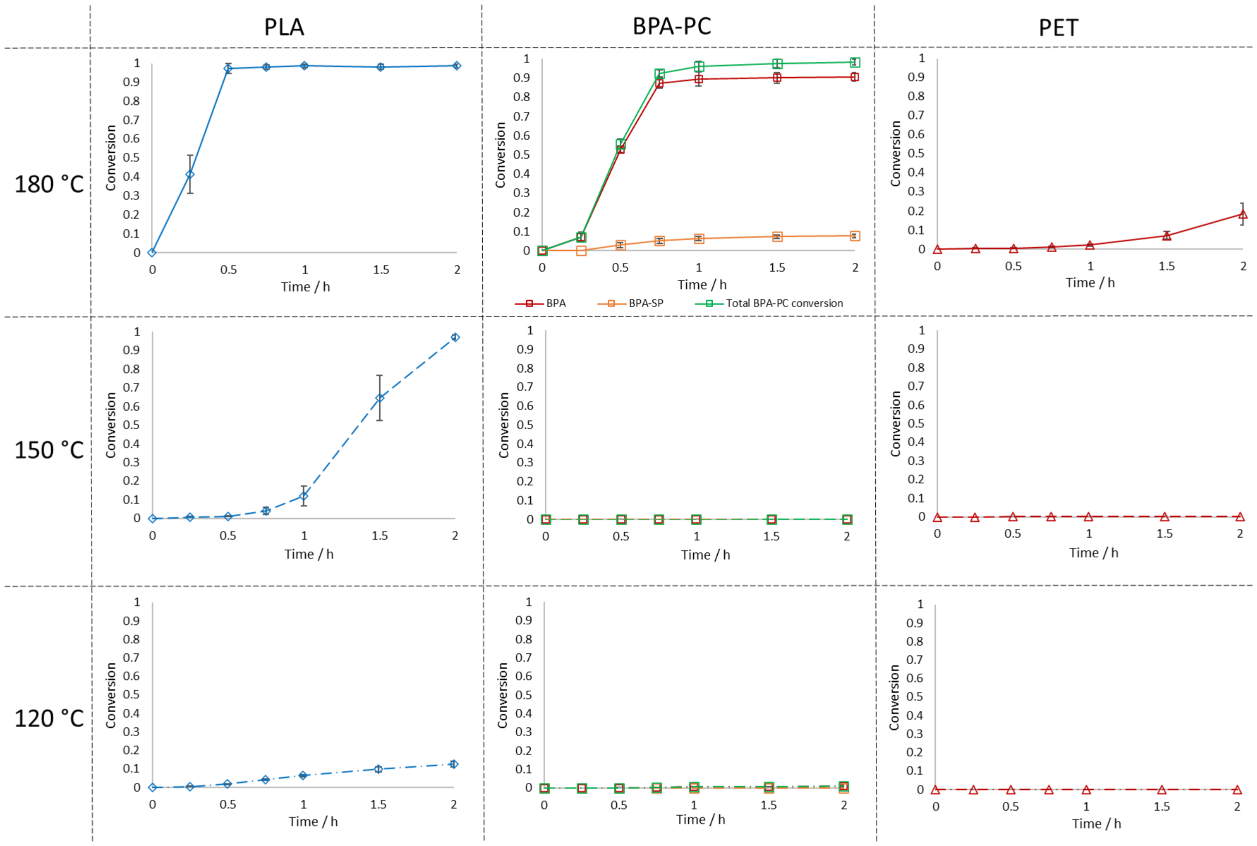

Figure S3. Kinetic plots of conversion against time for depolymerisation of PLA, BPA-PC and PET at 120 °C, 150 °C and 180 °C using magnesium chloride as single catalyst system. The kinetics were followed by ^{1}H NMR spectroscopy in DMSO-d_6. Conversions of PLA to 2-HEtLa (δ_H = 1.24 (d, 3H)), BPA-PC to BPA (δ_H = 6.90 (d, 4H), 6.65 (d, 4H)) and PET to BHET (δ_H = 8.10 (s, 4H)) were determined using NMP as an internal standard (δ_H = 2.71 (s, 3H)).

DMAP

Following the general procedure, the kinetics of the depolymerization of PLA, BPA-PC and PET in EG using 0.15 equiv. of DMAP single catalyst system was conducted at 120 °C, 150 °C and 180 °C. Total BPA-PC conversion was taken as the sum of conversion to BPA and conversion to BPA-SP.

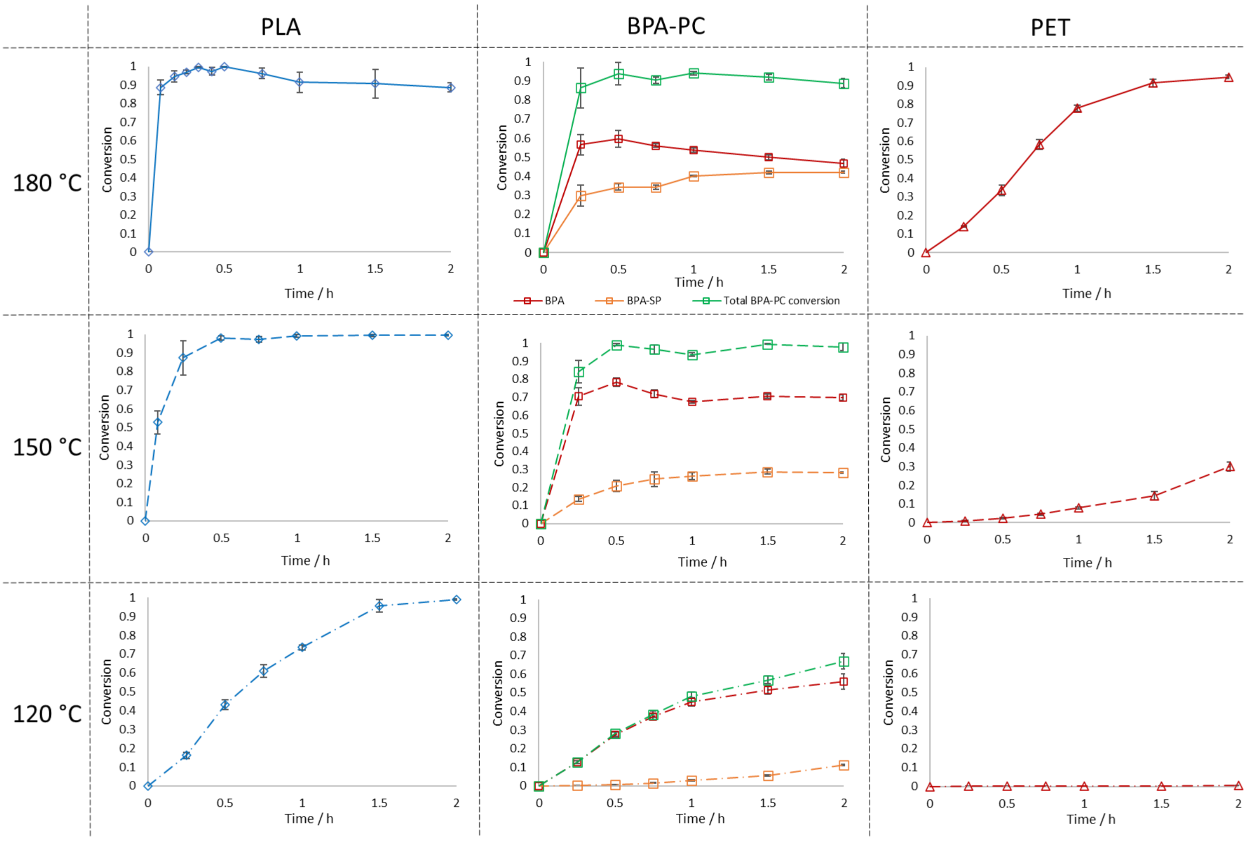

Figure S4. Kinetic plots of conversion against time for depolymerisation of PLA, BPA-PC and PET at 120 °C, 150 °C and 180 °C using DMAP as single catalyst system. The kinetics were followed by ^{1}H NMR spectroscopy in DMSO-d_6. Conversions of PLA to 2-HEtLa (δ_H = 1.24 (d, 3H)), BPA-PC to BPA (δ_H = 6.90 (d, 4H), 6.65 (d, 4H)) and PET to BHET (δ_H = 8.10 (s, 4H)) were determined using NMP as an internal standard (δ_H = 2.71 (s, 3H)).

Imidazole

Following the general procedure, the kinetics of the depolymerization of PLA, BPA-PC and PET in EG using 0.15 equiv. of imidazole single catalyst system was conducted at 120 °C, 150 °C and 180 °C. Total BPA-PC conversion was taken as the sum of conversion to BPA and conversion to BPA-SP.

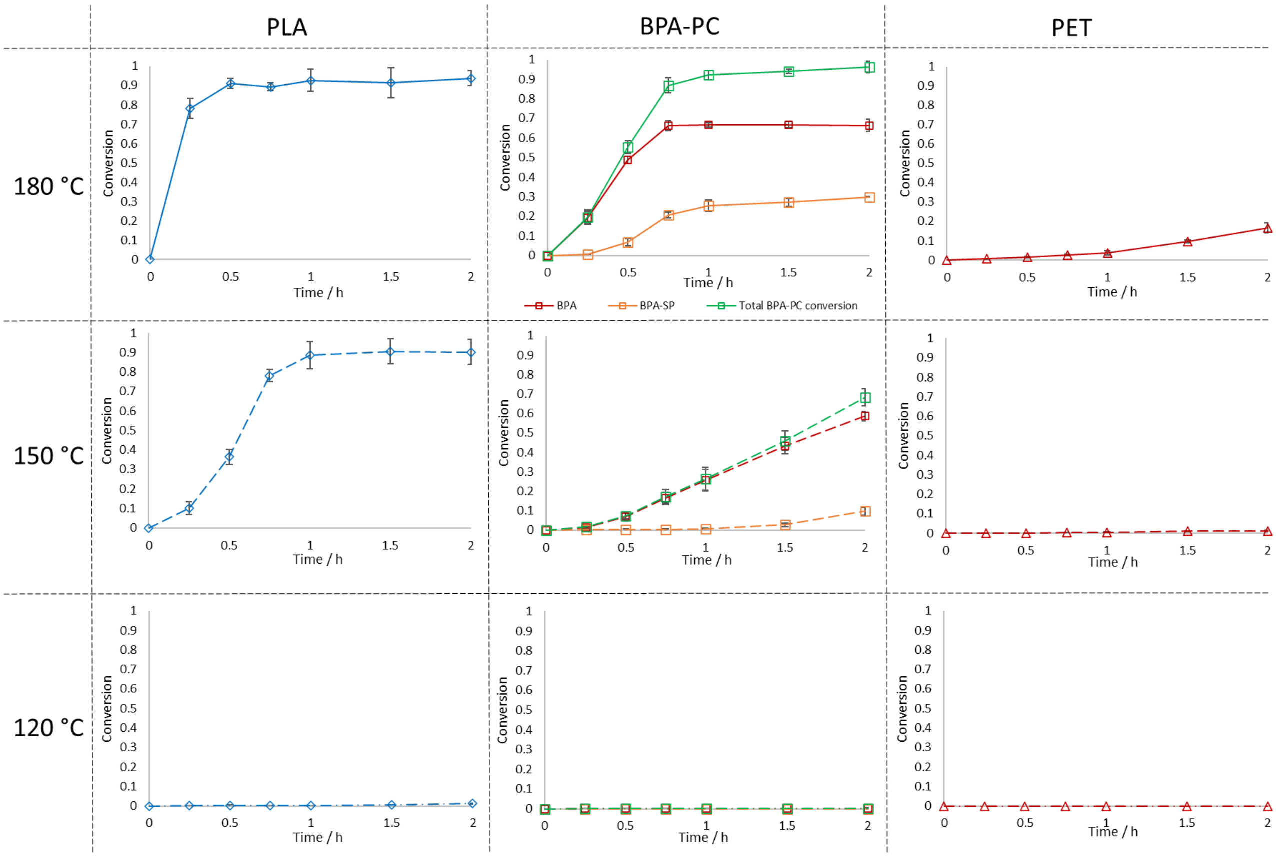

Figure S5. Kinetic plots of conversion against time for depolymerisation of PLA, BPA-PC and PET at 120 °C, 150 °C and 180 °C using imidazole as single catalyst system. The kinetics were followed by ^{1}H NMR spectroscopy in DMSO-d_6. Conversions of PLA to 2-HEtLa (δ_H = 1.24 (d, 3H)), BPA-PC to BPA (δ_H = 6.90 (d, 4H), 6.65 (d, 4H)) and PET to BHET (δ_H = 8.10 (s, 4H)) were determined using NMP as an internal standard (δ_H = 2.71 (s, 3H)).

Kinetic studies of PLA, BPA-PC and PET depolymerization using dual catalyst systems

Zn(OAc)$_2$·2H$_2$O / DMAP

Following the general procedure, the kinetics of the depolymerization of PLA, BPA-PC and PET in EG using 0.15 equiv. of Zn(OAc)$_2$ / DMAP dual catalyst system was conducted at 120 °C, 150 °C and 180 °C. Total BPA-PC conversion was taken as the sum of conversion to BPA and conversion to BPA-SP.

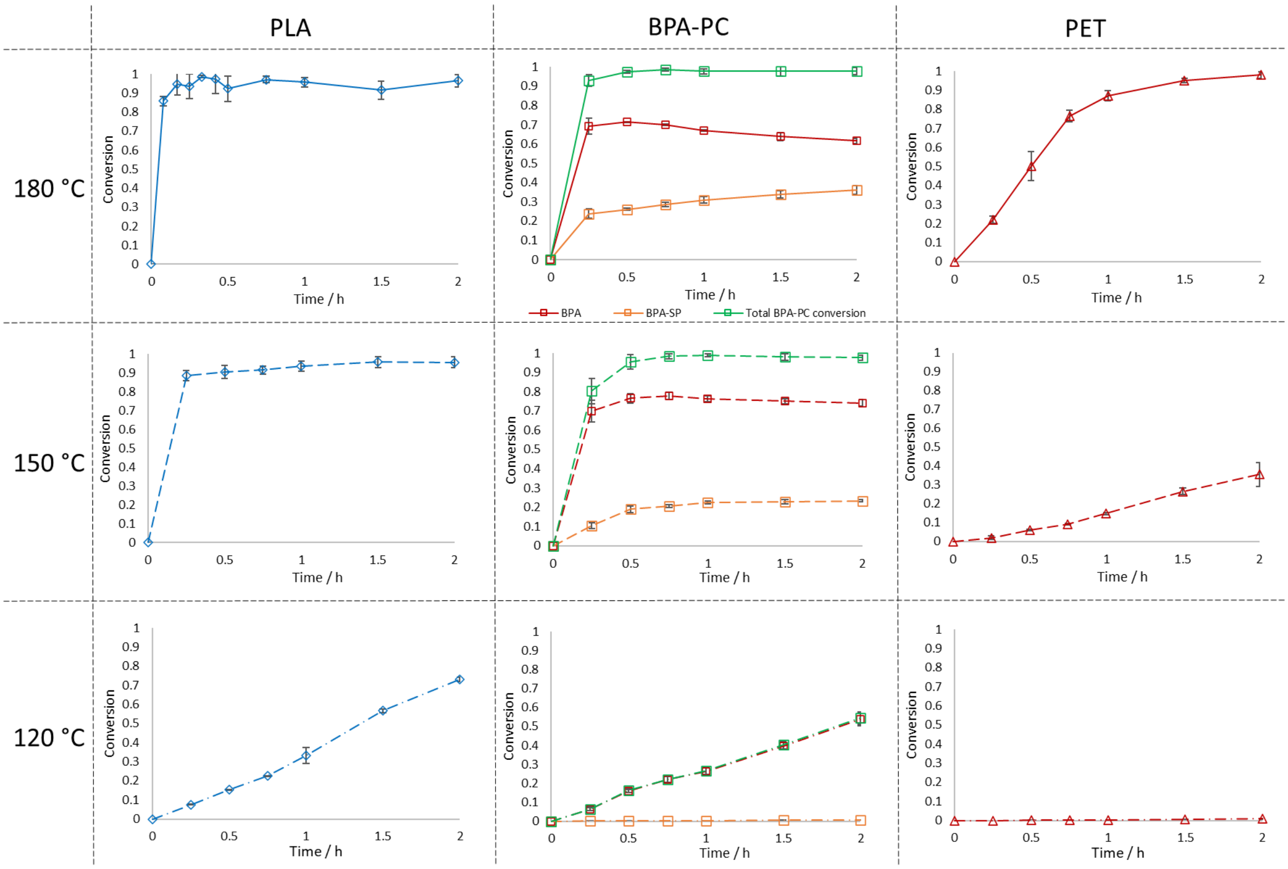

Figure S6. Kinetic plots of conversion against time for depolymerisation of PLA, BPA-PC and PET at 120 °C, 150 °C and 180 °C using Zn(OAc)$_2$·2H$_2$O / DMAP as dual catalyst system. The kinetics were followed by ^{1}H NMR spectroscopy in DMSO-d_6. Conversions of PLA to 2-HEtLa (δ_H = 1.24 (d, 3H)), BPA-PC to BPA (δ_H = 6.90 (d, 4H), 6.65 (d, 4H)) and PET to BHET (δ_H = 8.10 (s, 4H)) were determined using NMP as an internal standard (δ_H = 2.71 (s, 3H)).

Zn(OAc)$_2$·2H$_2$O / Imidazole

Following the general procedure, the kinetics of the depolymerization of PLA, BPA-PC and PET in EG using 0.15 equiv. of Zn(OAc)$_2$ / imidazole dual catalyst system was conducted at 120 °C, 150 °C and 180 °C. Total BPA-PC conversion was taken as the sum of conversion to BPA and conversion to BPA-SP.

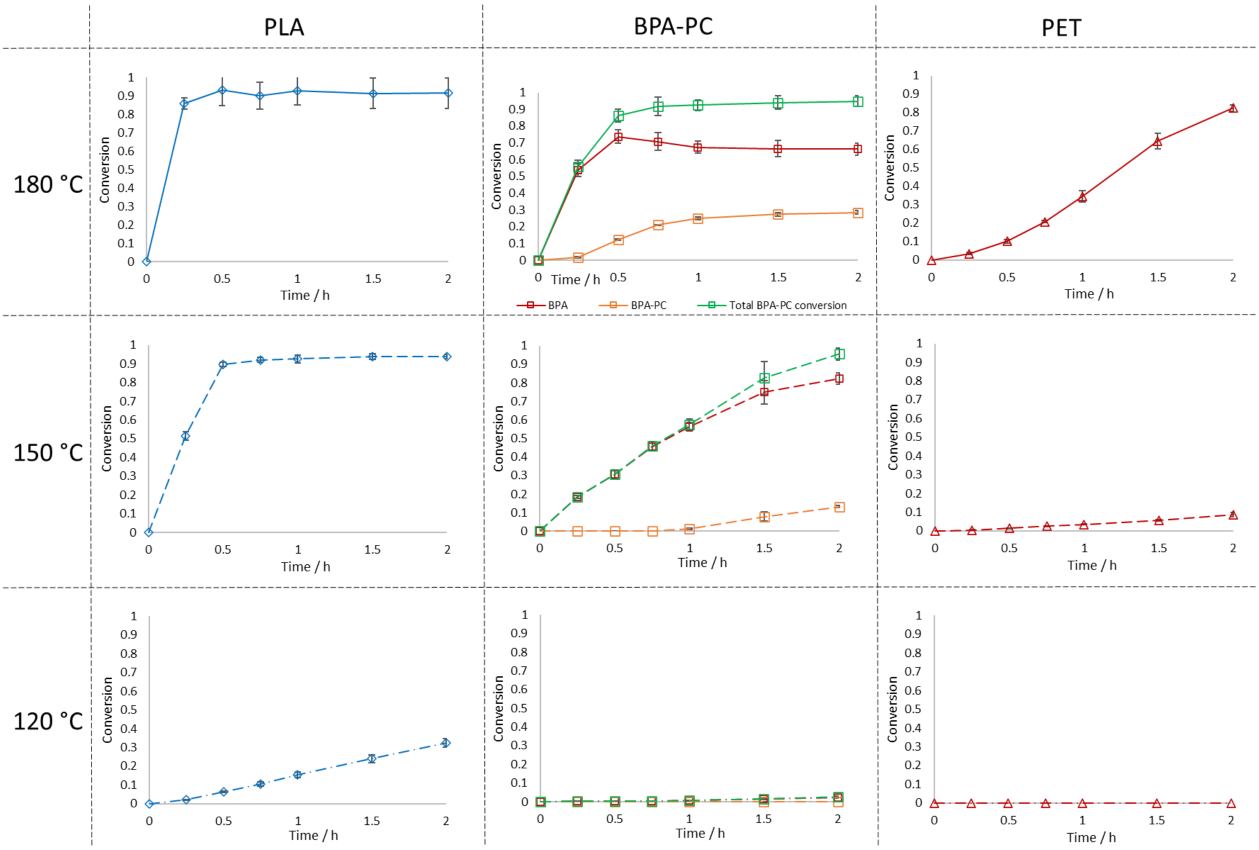

Figure S7. Kinetic plots of conversion against time for depolymerisation of PLA, BPA-PC and PET at 120 °C, 150 °C and 180 °C using Zn(OAc)$_2$·2H$_2$O / imidazole as dual catalyst system. The kinetics were followed by ^{1}H NMR spectroscopy in DMSO-d_6. Conversions of PLA to 2-HEtLa (δ_H = 1.24 (d, 3H)), BPA-PC to BPA (δ_H = 6.90 (d, 4H), 6.65 (d, 4H)) and PET to BHET (δ_H = 8.10 (s, 4H)) were determined using NMP as an internal standard (δ_H = 2.71 (s, 3H)).

$Mg(OAc)_2 \cdot 4H_2O$ / DMAP

Following the general procedure, the kinetics of the depolymerization of PLA, BPA-PC and PET in EG using 0.15 equiv. of $Mg(OAc)_2$ / DMAP dual catalyst system was conducted at 120 °C, 150 °C and 180 °C. Total BPA-PC conversion was taken as the sum of conversion to BPA and conversion to BPA-SP.

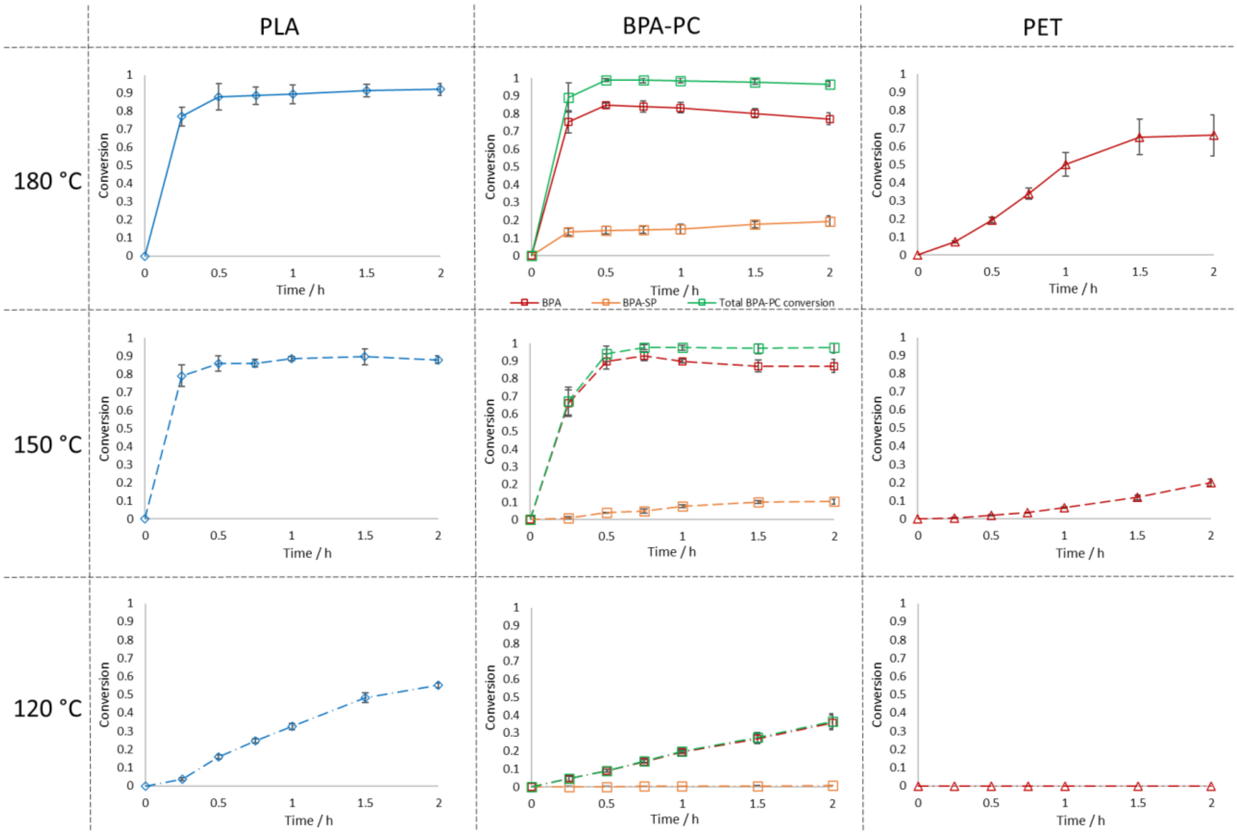

Figure S8. Kinetic plots of conversion against time for depolymerisation of PLA, BPA-PC and PET at 120 °C, 150 °C and 180 °C using $Mg(OAc)_2 \cdot 4H_2O$ / DMAP as dual catalyst system. The kinetics were followed by ^{1}H NMR spectroscopy in DMSO-d_6. Conversions of PLA to 2-HEtLa (δ_H = 1.24 (d, 3H)), BPA-PC to BPA (δ_H = 6.90 (d, 4H), 6.65 (d, 4H)) and PET to BHET (δ_H = 8.10 (s, 4H)) were determined using NMP as an internal standard (δ_H = 2.71 (s, 3H)).

$Mg(OAc)_2·4H_2O$ / Imidazole

Following the general procedure, the kinetics of the depolymerization of PLA, BPA-PC and PET in EG using 0.15 equiv. of $Mg(OAc)_2$ / imidazole dual catalyst system was conducted at 120 °C, 150 °C and 180 °C. Total BPA-PC conversion was taken as the sum of conversion to BPA and conversion to BPA-SP.

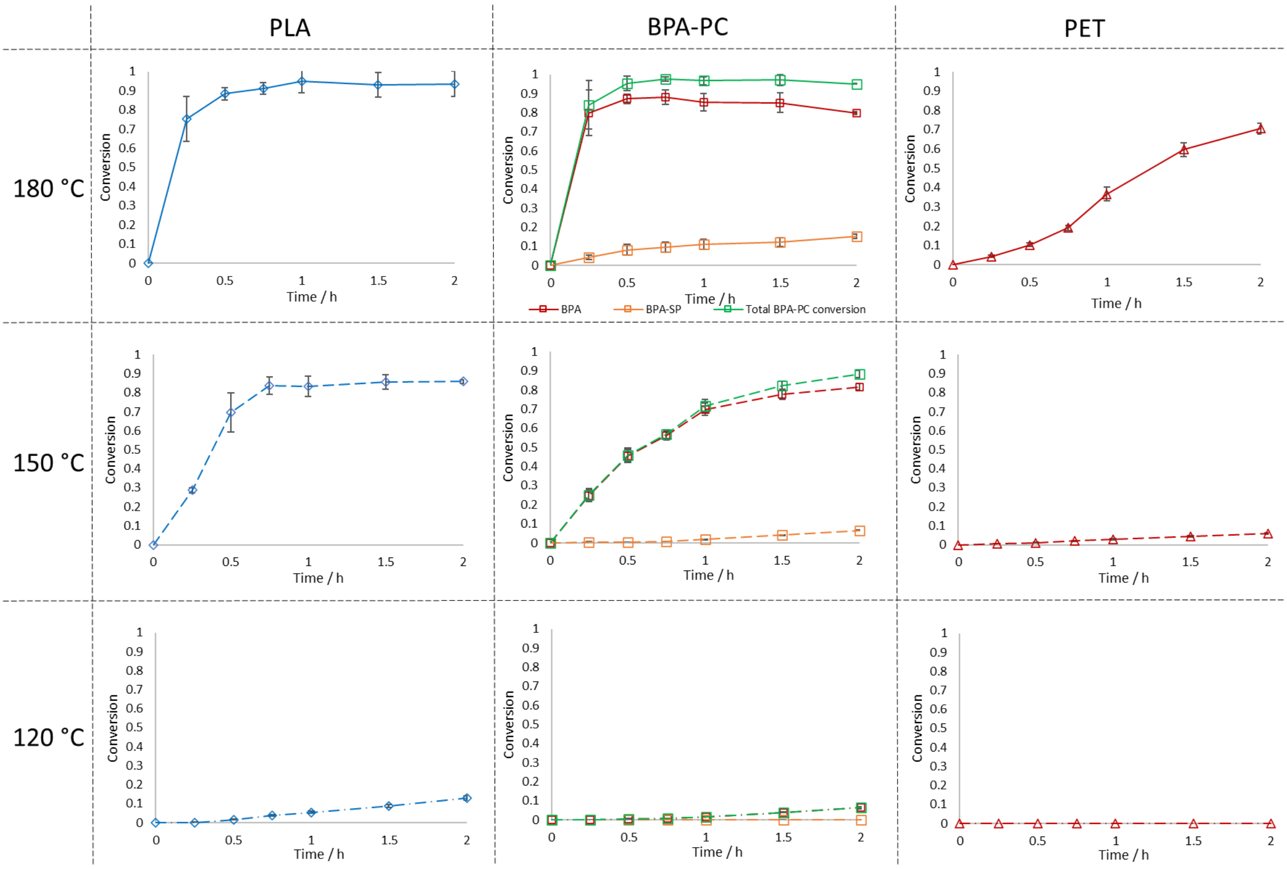

Figure S9. Kinetic plots of conversion against time for depolymerisation of PLA, BPA-PC and PET at 120 °C, 150 °C and 180 °C using $Mg(OAc)_2·4H_2O$ / imidazole as dual catalyst system. The kinetics were followed by ^1H NMR spectroscopy in DMSO-d_6. Conversions of PLA to 2-HEtLa (δ_H = 1.24 (d, 3H)), BPA-PC to BPA (δ_H = 6.90 (d, 4H), 6.65 (d, 4H)) and PET to BHET (δ_H = 8.10 (s, 4H)) were determined using NMP as an internal standard (δ_H = 2.71 (s, 3H)).

$MgCl_2$ / DMAP

Following the general procedure, the kinetics of the depolymerization of PLA, BPA-PC and PET in EG using 0.15 equiv. of $MgCl_2$ / DMAP dual catalyst system was conducted at 120 °C, 150 °C and 180 °C. Total BPA-PC conversion was taken as the sum of conversion to BPA and conversion to BPA-SP.

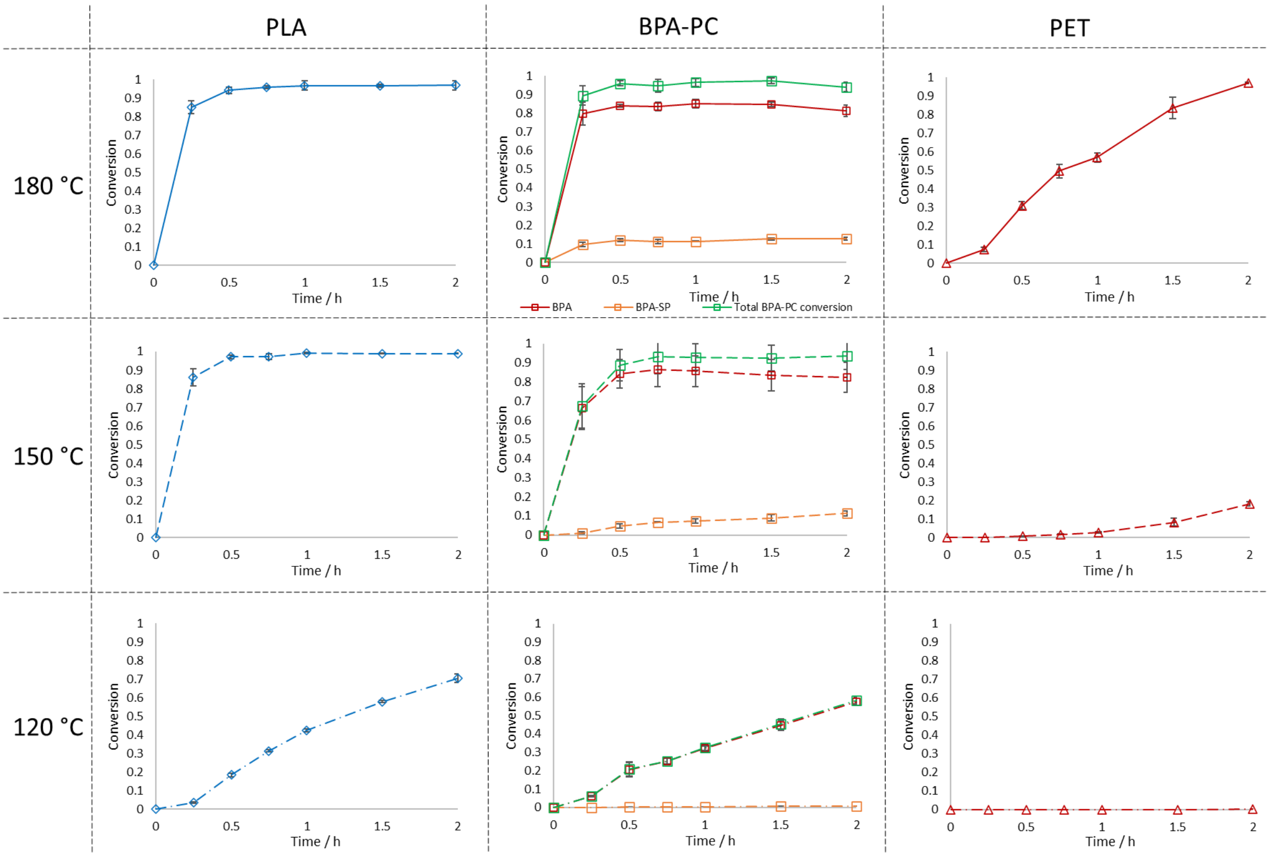

Figure S10. Kinetic plots of conversion against time for depolymerisation of PLA, BPA-PC and PET at 120 °C, 150 °C and 180 °C using $MgCl_2$/ DMAP as dual catalyst system. The kinetics were followed by ^{1}H NMR spectroscopy in DMSO-d_6. Conversions of PLA to 2-HEtLa (δ_H = 1.24 (d, 3H)), BPA-PC to BPA (δ_H = 6.90 (d, 4H), 6.65 (d, 4H)) and PET to BHET (δ_H = 8.10 (s, 4H)) were determined using NMP as an internal standard (δ_H = 2.71 (s, 3H)).

$MgCl_2$ / Imidazole

Following the general procedure, the kinetics of the depolymerization of PLA, BPA-PC and PET in EG using 0.15 equiv. of $MgCl_2$ / imidazole dual catalyst system was conducted at 120 °C, 150 °C and 180 °C. Total BPA-PC conversion was taken as the sum of conversion to BPA and conversion to BPA-SP.

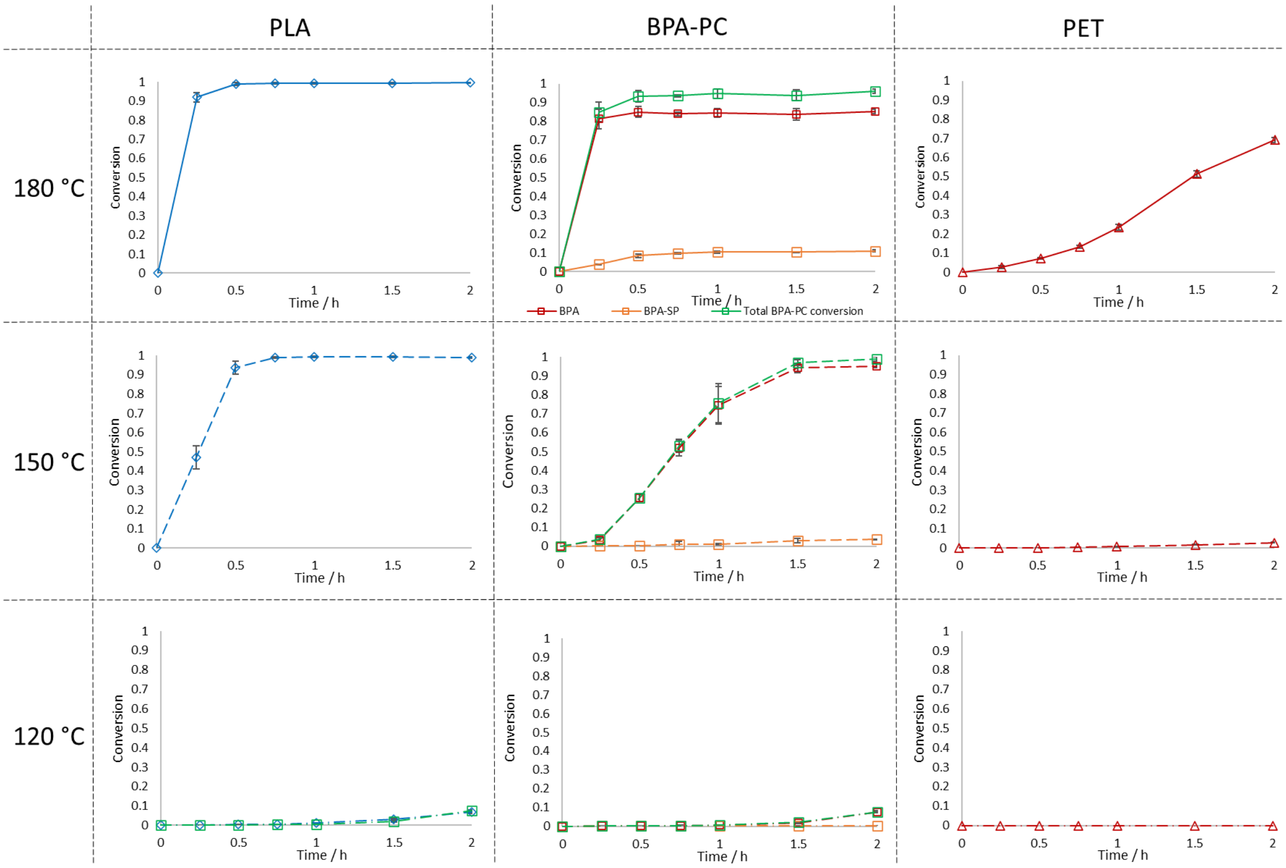

Figure S11. Kinetic plots of conversion against time for depolymerisation of PLA, BPA-PC and PET at 120 °C, 150 °C and 180 °C using $MgCl_2$/ imidazole as dual catalyst system. The kinetics were followed by ^{1}H NMR spectroscopy in DMSO-d_6. Conversions of PLA to 2-HEtLa (δ_H = 1.24 (d, 3H)), BPA-PC to BPA (δ_H = 6.90 (d, 4H), 6.65 (d, 4H)) and PET to BHET (δ_H = 8.10 (s, 4H)) were determined using NMP as an internal standard (δ_H = 2.71 (s, 3H)).

Optimized sole PLA depolymerization to 2-HEtLa

Following the general procedure, PLA (2.5 mmol), $MgCl_2$ (0.15 equiv.), ethylene glycol (50 mmol) were charged in a 20 mL vial equipped with a magnetic stirrer. The reaction was heated to 150 °C and checked every 30 minutes until complete depolymerization. After cooling down, the reaction mixture was diluted with DCM, and washed with brine. The organic layer was dried over $MgSO_4$, filtered and concentrated under reduced pressure to give 2-HEtLa with spectroscopic data in accordance with the literature.[1]

^{1}H NMR ($CDCl_3$, 400 MHz) δ_H = 4.35 – 4.19 (m, 3H), 3.81 (dd, J = 4.9, 4.3 Hz, 2H), 3.68 (s, 1H), 1.40 (d, J = 6.9 Hz, 3H).

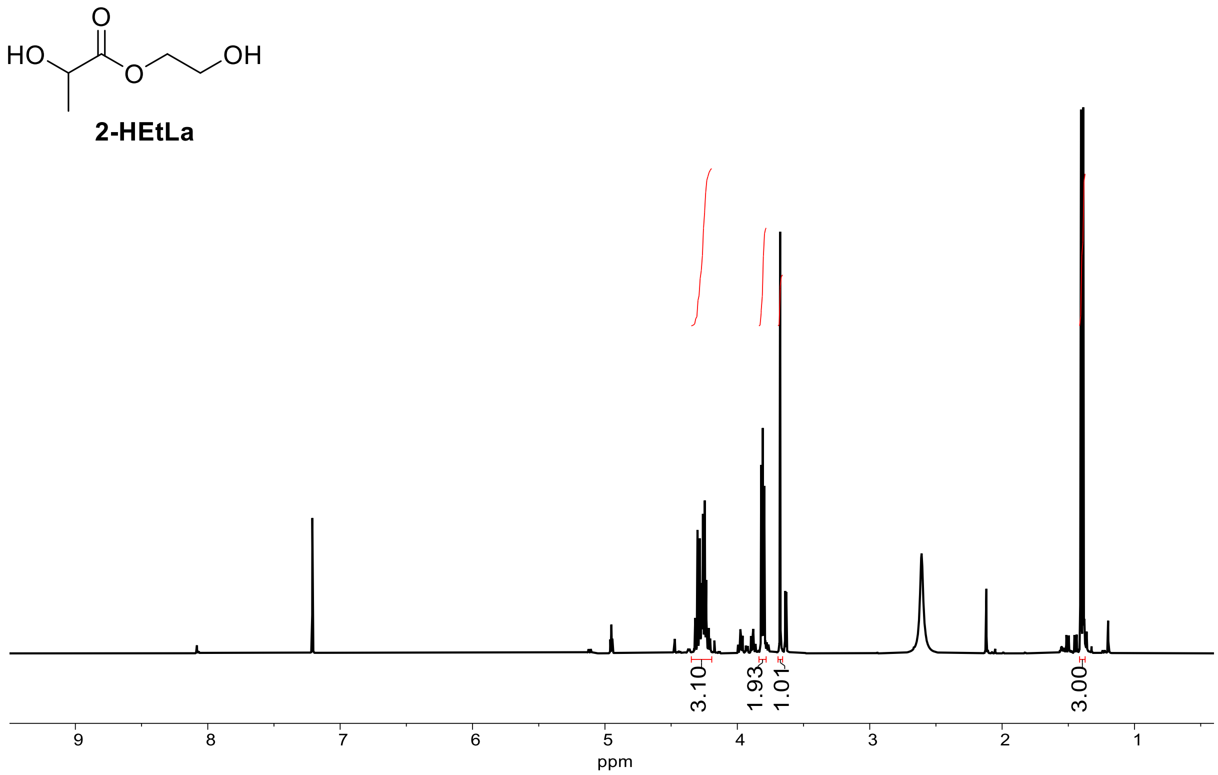

Figure S12. ^{1}H NMR ($CDCl_3$, 400 MHz) spectra of 2-HEtLa.

Optimized sole BPA-PC depolymerization to BPA

Following the general procedure, BPA-PC (2.5 mmol), $MgCl_2$ / imidazole (0.15 equiv.), ethylene glycol (50 mmol) were charged in a 20 mL vial equipped with a magnetic stirrer. The reaction was heated to 150 ºC and checked every 30 minutes until complete depolymerization.

After cooling down, the reaction mixture was diluted with DCM, and washed with brine. The organic layer was dried over $MgSO_4$, filtered and concentrated under reduced pressure The crude product was purified by column chromatography (60:40 Hexane: Ethyl Acetate) to give BPA and ethylene carbonate with spectroscopic data in accordance with the literature.[2]

BPA, ^{1}H NMR (DMSO-d_6, 400 MHz) δ_H = 9.14 (s, 2H), 7.05 – 6.89 (m, 4H), 6.72 – 6.54 (m, 4H), 1.53 (s, 6H).
EC, ^{1}H NMR (DMSO-d_6, 400 MHz) δ_H = 4.51 – 4.42 (m, 4H).

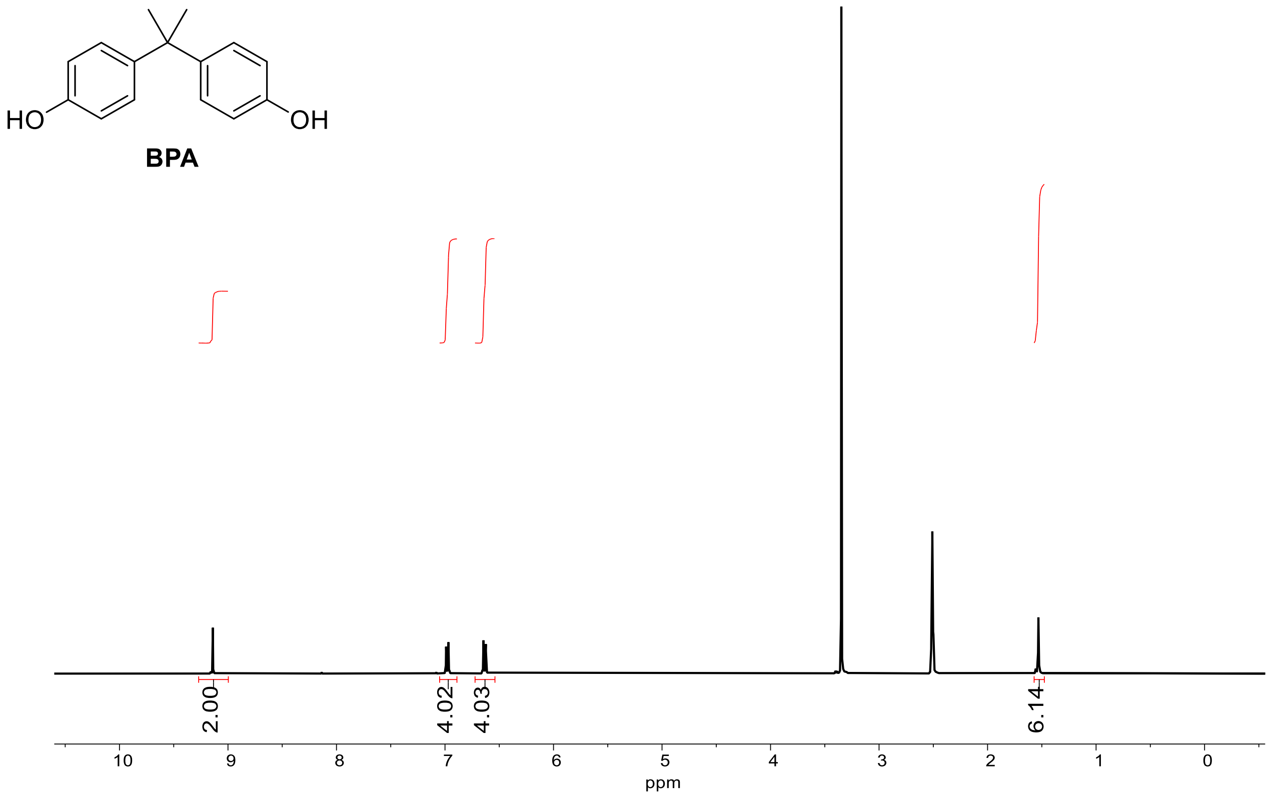

Figure 13. ^{1}H-NMR (DMSO-d_6, 400 MHz) spectra of the isolated BPA.

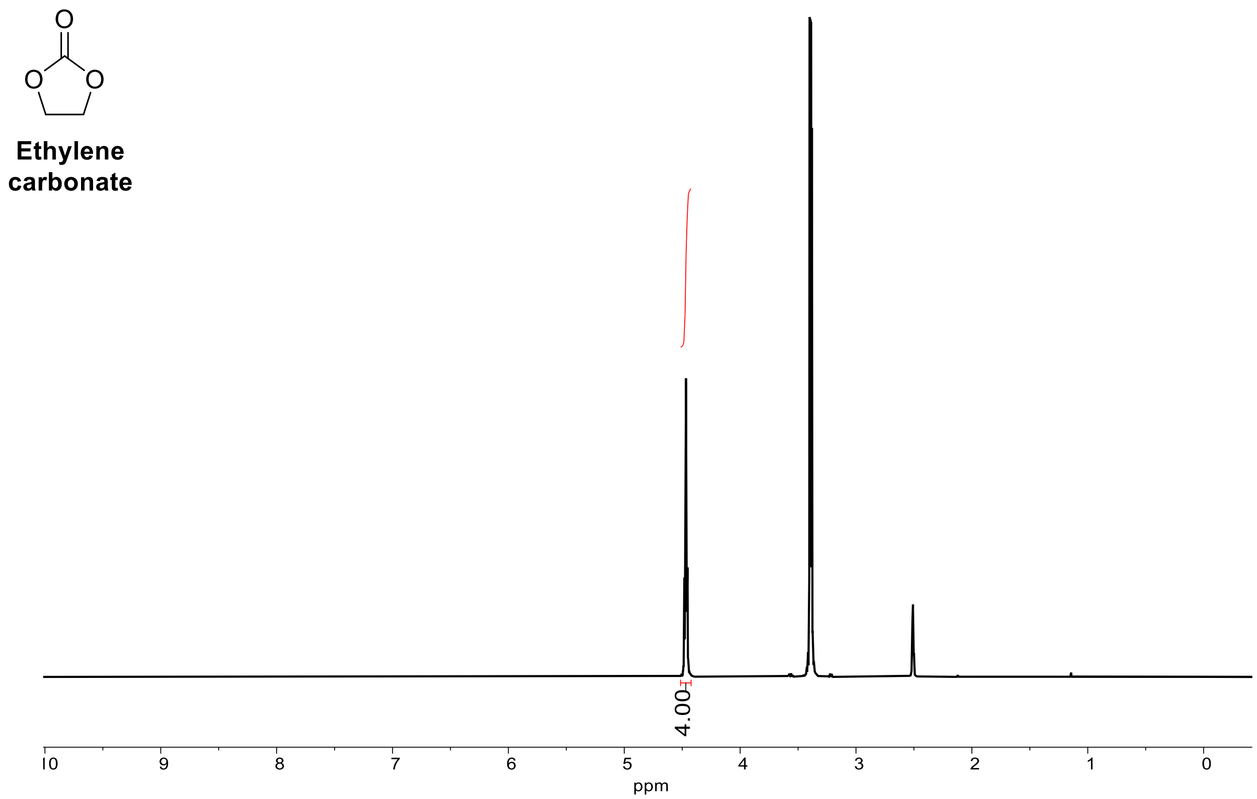

Figure S14. ^{1}H-NMR (DMSO-d_6, 400 MHz) spectra of the isolated ethylene carbonate.

Optimized sole PET depolymerization to BHET

Following the general procedure, PET (2.5 mmol) $Mg(OAc)_2$ / imidazole (0.15 equiv.), ethylene glycol (50 mmol) were charged in a 20 mL vial equipped with a magnetic stirrer. The reaction was heated to 180 ºC and checked every 30 minutes until complete depolymerization.

BHET was isolated after precipitation from cold water as a white solid with spectroscopic data in accordance with the literature.[2,3]

^{1}H NMR (DMSO-d_6, 400 MHz) δ_H = 8.14 (s, 4H), 4.98 (t, J = 5.7 Hz, 2H), 4.33 (t, 4H), 3.73 (q, 4H).

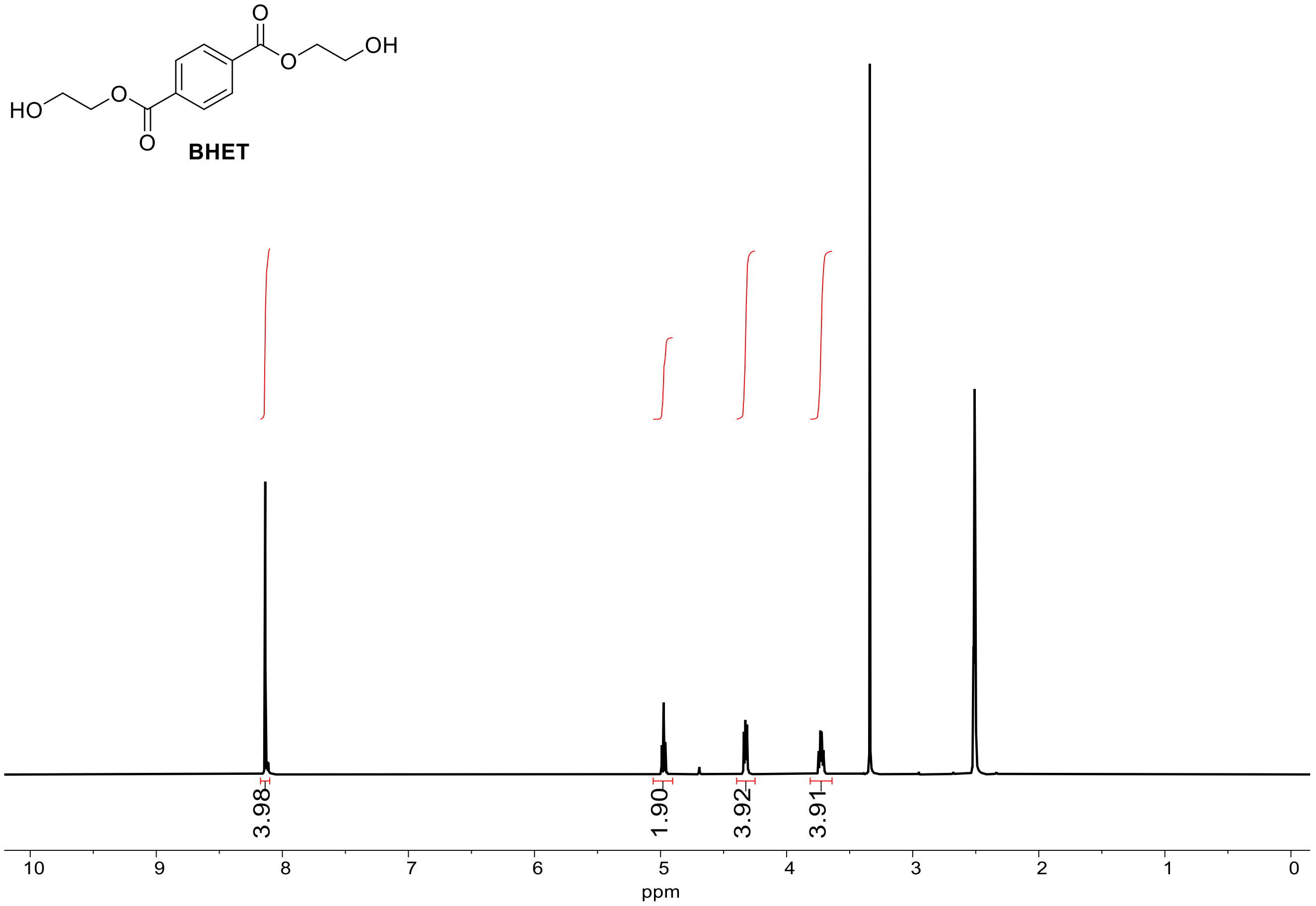

Figure S15. ^{1}H-NMR (DMSO-d_6, 400 MHz) spectra of the isolated BHET.

Kinetic studies for the selective PLA, BPA-PC and PET depolymerization using single and dual catalyst systems

Following the above-reported procedure, the sequential selective depolymerization of PLA, BPA-PC and PET was conducted using EG as nucleophile.

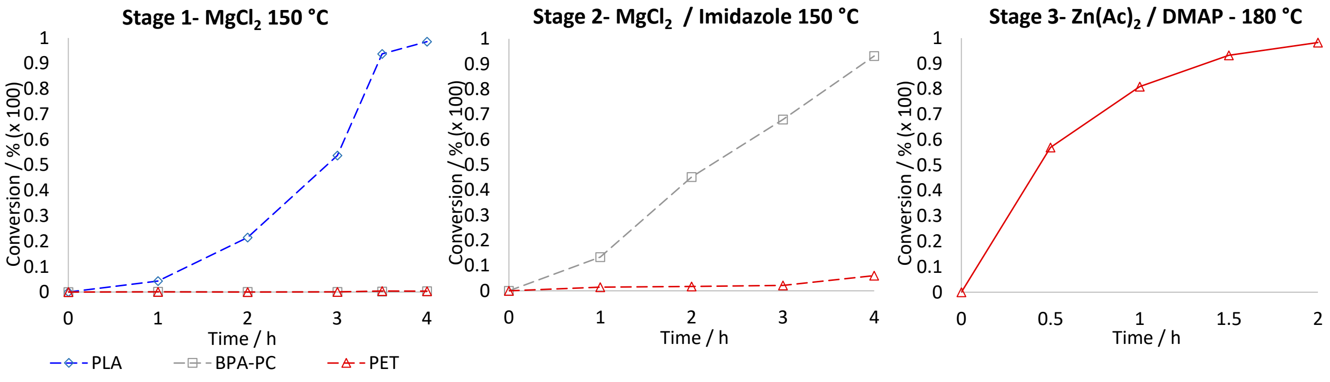

Figure S16. Kinetic plots of conversion against time for the selective and sequential depolymerisation of PLA, BPA-PC and PET using both single and dual catalyst system. The kinetics were followed by ^{1}H NMR spectroscopy in DMSO-d_6 using NMP as internal standard.

Sequential selective depolymerisation reactions in presence of ethylene glycol

Step 1 – Selective glycolysis of PLA

A 20 mL scintillation vial with a magnetic stirrer bead was charged with magnesium chloride (0.375 mmol, 15 mol%), ethylene glycol (50 mmol, 2000 mol%) and the internal standard NMP (0.25 mmol, 10 mol%). The vial was sealed and placed within a heating block at 150 °C, whilst stirring at 500 rpm, for 15 minutes to solubilise the catalyst and allow the reaction mixture temperature to stabilise. After 15 minutes, an aliquot (0.1 mL) was removed from the reaction mixture to act as a t_0 for NMR spectroscopy analysis, pelletised PLA, BPA-PC and PET (2.5 mmol of each) were then added, the vial was resealed, and a timer set. Further aliquots for NMR spectroscopy analysis were taken at t = 1 h, 2 h, 3 h, 3.5 h and 4 h. All aliquots taken for ^{1}H NMR spectroscopy analysis were dissolved in DMSO-d_6. At reaction end, the vial was removed from the heating block and the magnetic stirrer was removed. Unreacted PET and BPA-PC pellets were removed from the reaction mixture, washed with ethylene glycol, dried, and then weighed before usage in the following stage. The vial containing the reaction mixture was sealed and placed in the fridge to cool down overnight, prior to recovery of 2-HEtLa.

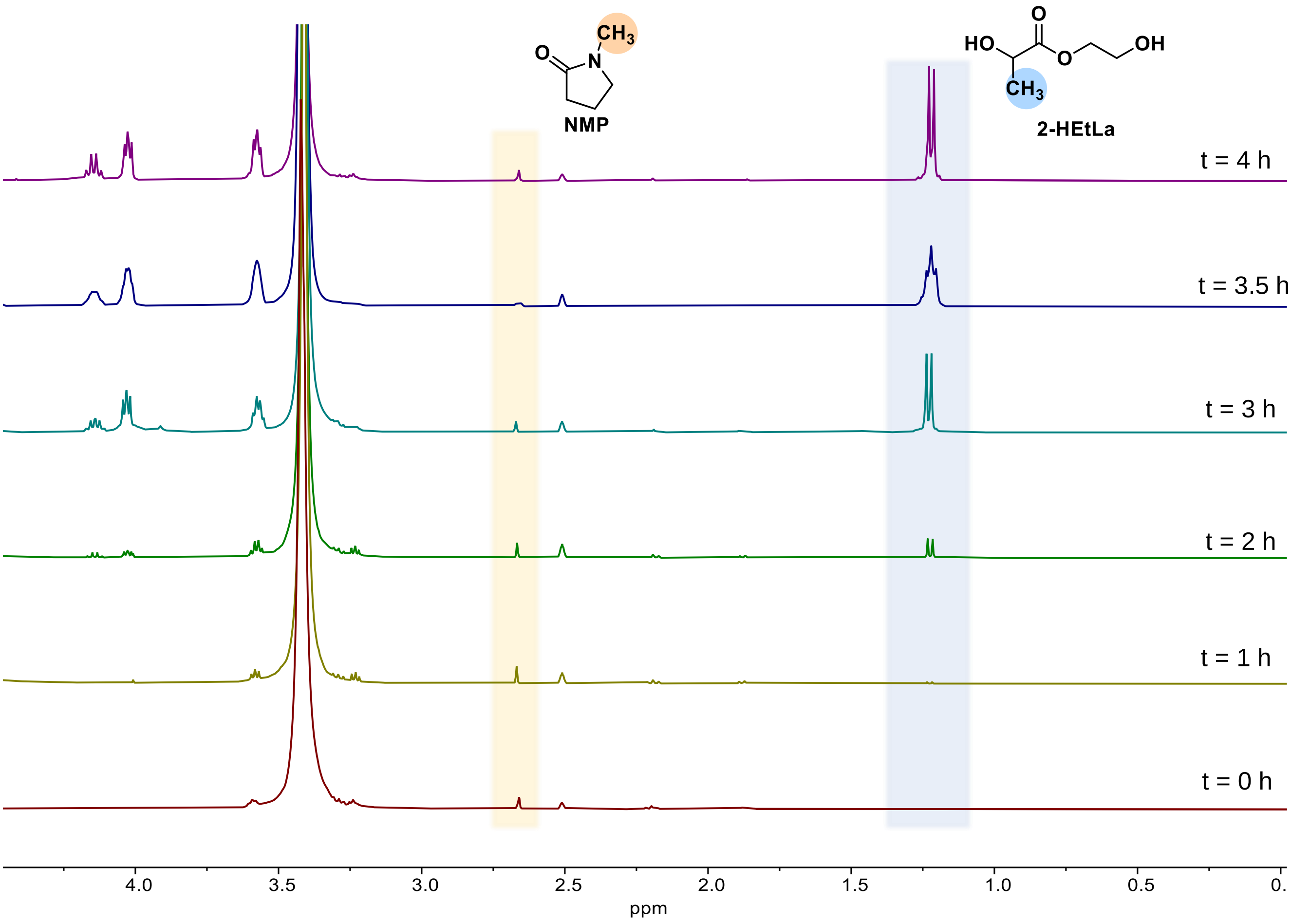

Figure S17. Stacked ^{1}H-NMR spectra of a selective depolymerisation reaction between PLA, BPA-PC and PET from t = 0 h to t = 4 h at 150 °C, using $MgCl_2$ as catalyst system, showing the peaks used to measure conversion of PLA to 2-HEtLa (δ_H = 1.24 (d, 3H)), in presence of NMP which was used as an internal standard (δ_H = 2.71 (s, 3H)).

Step 2 – Selective depolymerisation of BPA-PC by glycolysis

A 20 mL scintillation vial with a magnetic stirrer bead was charged with magnesium chloride and imidazole (0.375 mmol, 15 mol% of each), ethylene glycol (50 mmol, 2000 mol%) and the internal standard NMP (0.25 mmol, 10 mol%). The vial was sealed and placed within a heating block at 150 °C, whilst stirring at 500 rpm, for 15 minutes to solubilise the catalyst. An aliquot (0.1 mL) was later removed from the reaction mixture to act as a t_0 for NMR spectroscopy analysis, pelletised BPA-PC and PET from the previous stage were then added, and the vial was resealed. Further aliquots for NMR spectroscopy analysis were taken at t = 1 h, 2 h, 3 h and 4 h. All aliquots taken for ^{1}H NMR spectroscopy analysis were dissolved in DMSO-d_6. At reaction end, the vial was removed from the heating block and the magnetic stirrer was removed. Unreacted PET pellets were removed from the reaction mixture, washed with ethylene glycol, dried, and then weighed before usage in the following stage. The vial containing the reaction mixture was sealed and placed in the fridge to cool down overnight, prior to recovery of BPA, ethylene carbonate.

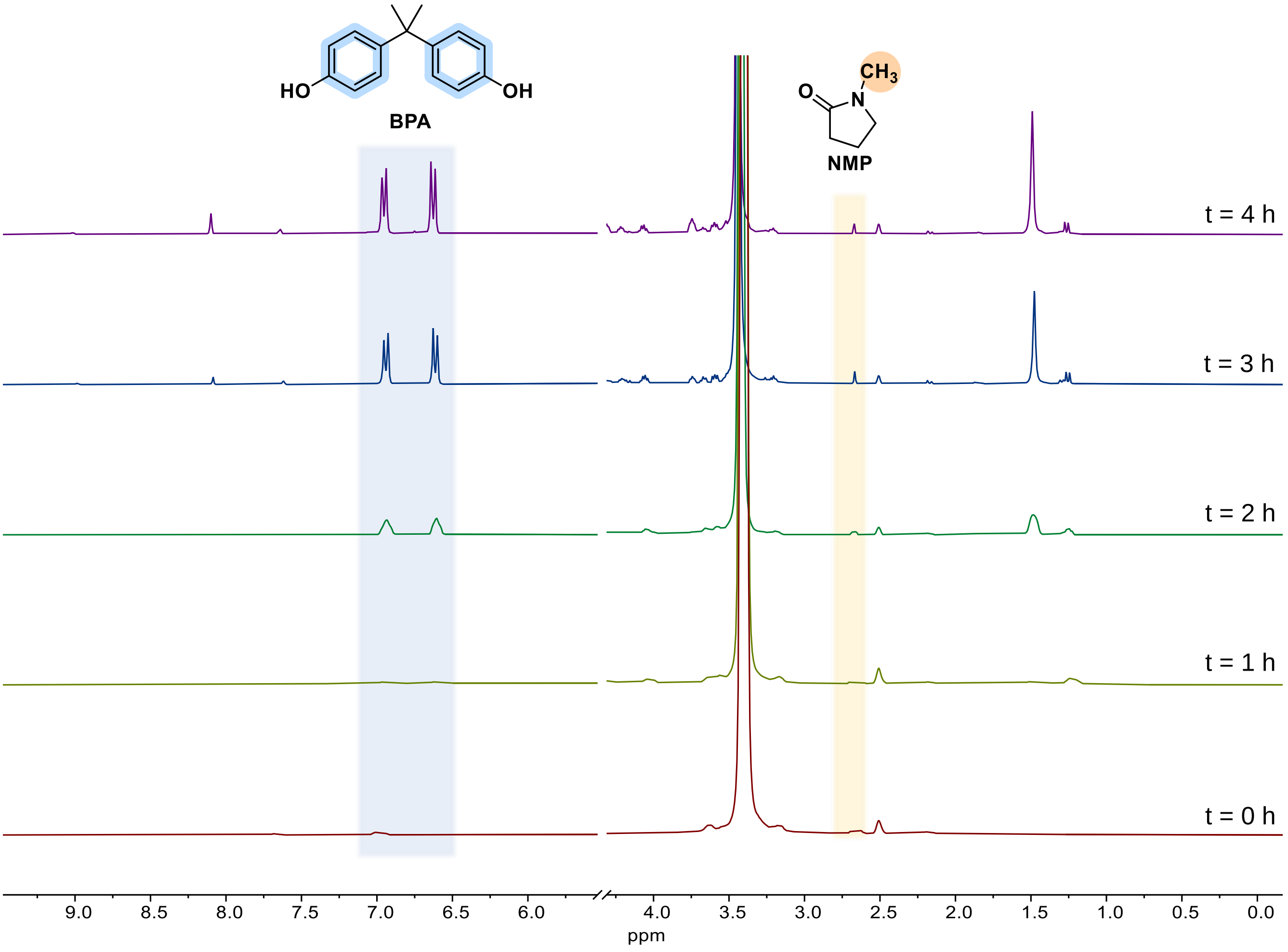

Figure S18. Stacked ^{1}H-NMR spectra of a selective depolymerisation reaction between BPA-PC and PET from t = 0 h to t = 4 h at 150 °C, using $MgCl_2$ / imidazole as catalyst system, showing the peaks used to measure conversion of BPA-PC to BPA (δ_H = 6.90 (d, 4H), 6.65 (d, 4H)) and PET to BHET (δ_H = 8.10 (s, 4H)), alongside that of NMP which was used as an internal standard (δ_H = 2.71 (s, 3H)).

Step 3 – Depolymerisation of PET by glycolysis

A 20 mL scintillation vial with a magnetic stirrer bead was charged with zinc acetate dihydrate and DMAP (0.375 mmol, 15 mol% of each), ethylene glycol (50 mmol, 2000 mol%) and the internal standard NMP (0.25 mmol, 10 mol%). The vial was sealed and placed within a heating block at 180 °C, whilst stirring at 500 rpm, for 15 minutes to solubilise the catalyst and allow the reaction mixture temperature to stabilise. After 15 minutes, an aliquot (0.1 mL) was removed from the reaction mixture to act as a t_0 for NMR spectroscopy analysis, pelletised PET from the previous stage was then added, the vial was resealed, and a timer was set. Further aliquots for NMR spectroscopy analysis were taken at t = 0.5 h, 1 h, 1.5 h and 2 h. All aliquots taken for ^{1}H NMR spectroscopy analysis were dissolved in DMSO-d_6. At t = 2 h, the vial was removed from the heating block and the magnetic stirrer removed. Cold deionised water was added to the reaction vial to precipitate BHET, before the vial was sealed and placed in the fridge overnight to cool to recover BHET.

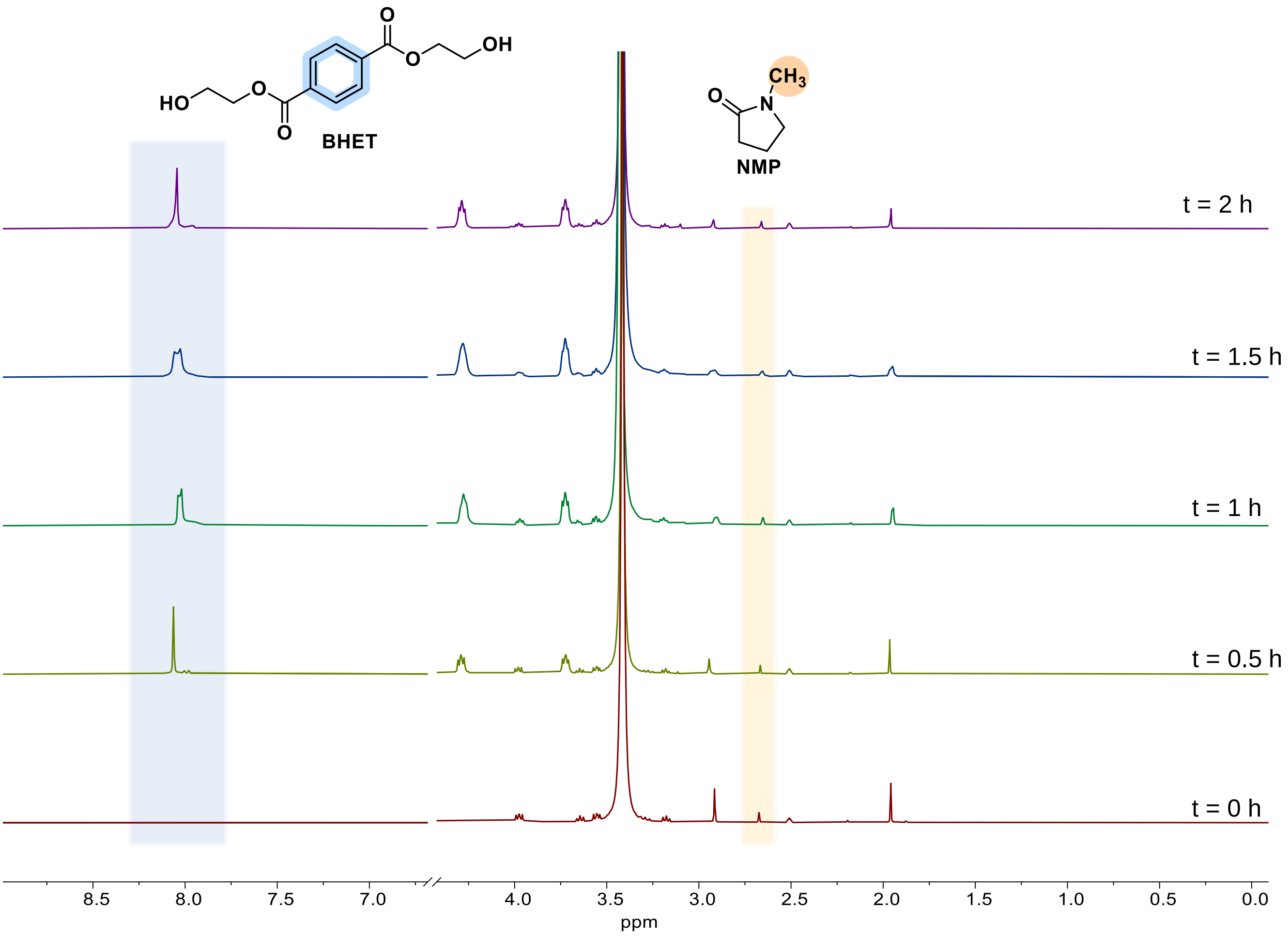

Figure S19. Stacked ^{1}H-NMR spectra of a PET glycolysis reaction from t = 0 h to t = 2 h at 180 °C, using $Zn(OAc)_2$ / DMAP as catalyst system, showing the peaks used to measure conversion to BHET (δ_H = 8.10 (s, 4H)), alongside that of NMP which was used as an internal standard (δ_H = 2.71 (s, 3H)).

Kinetic studies for selective depolymerization with alternative nucleophiles

A 20 mL scintillation vial with a magnetic stirrer bead was charged with magnesium chloride (0.375 mmol, 15 mol%), the stated nucleophiles (50 mmol, 2000 mol%) and the internal standard NMP (0.25 mmol, 10 mol%). The vial was sealed and placed within a heating block at 150 °C, whilst stirring at 500 rpm, for 15 minutes to solubilise the catalyst and allow the reaction mixture temperature to stabilise. After 15 minutes, an aliquot (0.1 mL) was removed from the reaction mixture to act as a t_0 for NMR spectroscopy analysis, pelletised PLA, BPA-PC and PET (2.5 mmol of each) were then added and the vial was resealed. Further aliquots for NMR spectroscopy analysis were taken at 0.25 h, 0.5 h, 0.75 h and 1 h. All aliquots taken for ^{1}H NMR spectroscopy analysis were dissolved in DMSO-d_6.

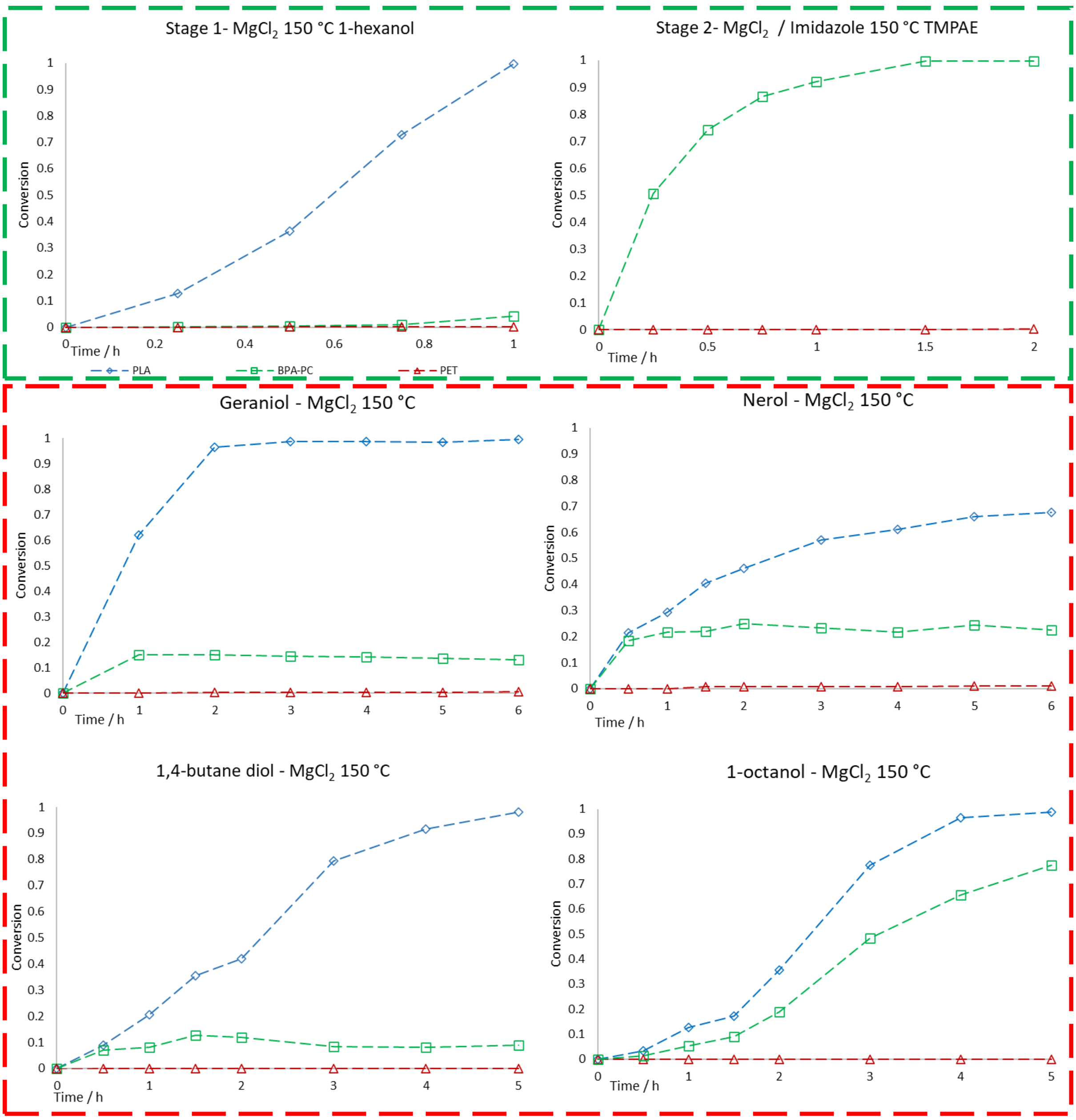

Figure S20. Kinetic plots of conversion against time for the mixed plastics PLA, PET and BPA-PC alcoholysis using alternative nucleophiles to enable chemical upcycling during selective depolymerisation reactions. Nucleophiles deemed appropriate for implementation in selective depolymerisation reactions are shown in the green box (top), whilst unsuccessful nucleophiles are shown in the red box (bottom).

Sequential selective depolymerisation reactions in presence of alternatives nucleophiles

Step 1 – Selective depolymerization of PLA in presence of 1-hexanol

A 20 mL scintillation vial with a magnetic stirrer bead was charged with magnesium chloride (0.375 mmol, 15 mol%), 1-hexanol (50 mmol, 2000 mol%) and the internal standard NMP (0.25 mmol, 10 mol%). The vial was sealed and placed within a heating block at 150 °C, whilst stirring at 500 rpm, for 15 minutes to solubilise the catalyst and allow the reaction mixture temperature to stabilise. After 15 minutes, an aliquot (0.1 mL) was removed from the reaction mixture to act as a t_0 for NMR spectroscopy analysis, pelletised PLA, BPA-PC and PET (2.5 mmol of each) were then added, the vial was resealed, and a timer set. Further aliquots for NMR spectroscopy analysis were taken at 0.25 h, 0.5 h, 0.75 h and 1 h. All aliquots taken for ^{1}H NMR spectroscopy analysis were dissolved in DMSO-d_6. At reaction end, the vial was removed from the heating block and the magnetic stirrer removed. Unreacted PET and BPA-PC pellets were removed from the reaction mixture, washed TMPAE (used as nucleophile in the subsequent step), dried, and then weighed before usage in the following stage. The vial containing the reaction mixture was sealed and placed in the fridge to cool down overnight, prior to recovery of hexyllactate.

Step 2 – Selective depolymerisation of BPA-PC in presence of TMPAE

A 20 mL scintillation vial with a magnetic stirrer bead was charged with magnesium chloride and imidazole (0.375 mmol, 15 mol% of each), ethylene glycol or TMPAE (50 mmol, 2000 mol%) and the internal standard NMP (0.25 mmol, 10 mol%). The vial was sealed and placed within a heating block at 150 °C, whilst stirring at 500 rpm, for 15 minutes to solubilise the catalyst and allow the reaction mixture temperature to stabilise. After 15 minutes, an aliquot (0.1 mL) was removed from the reaction mixture to act as a t_0 for NMR spectroscopy analysis, pelletised BPA-PC and PET from the previous stage were then added, the vial was resealed, and a timer set. Further aliquots for NMR spectroscopy analysis were taken at 0.25 h, 0.5 h, 0.75 h, 1 h, 1.5 h and 2 h. All aliquots taken for ^{1}H NMR spectroscopy analysis were dissolved in DMSO-d_6. At reaction end, the vial was removed from the heating block and the magnetic stirrer removed. Unreacted PET pellets were removed from the reaction mixture, washed with ethylene glycol, dried, and then weighed before usage in the following stage. The vial containing the reaction mixture was sealed and placed in the fridge to cool down overnight, prior to recovery of BPA and AOMEC.

Comparisons of Time/ Conversion plots for each step of the three selective depolymerisation routes

Following the general procedures above reported the sequential selective depolymerization of PLA, BPA-PC and PET was explored using alternative nucleophiles in the different steps.

Route 1

PLA, BPA-PC, PET —Step 1 (Ethylene glycol)→ BPA-PC, PET —Step 2 (Ethylene glycol)→ PET —Step 3 (Ethylene glycol)→ BHET recovered

(2-hydroxyethyl lactate recovered) (BPA and ethylene carbonate recovered)

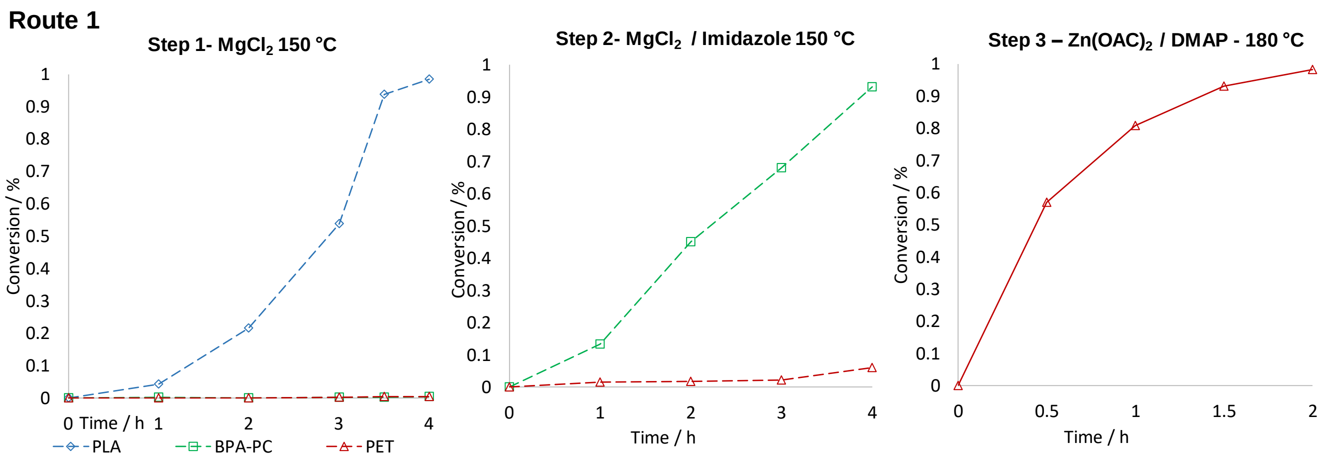

Figure S21. Kinetic plots of conversion against time for the selective and sequential depolymerisation for each step of the Route 1 selective depolymerisation.

Route 2

PLA, BPA-PC, PET —Step 1 (Ethylene glycol)→ BPA-PC, PET —Step 2 (**TMPAE**)→ PET —Step 3 (Ethylene glycol)→ BHET recovered

(2-hydroxyethyl lactate recovered) (BPA and AOEMC)

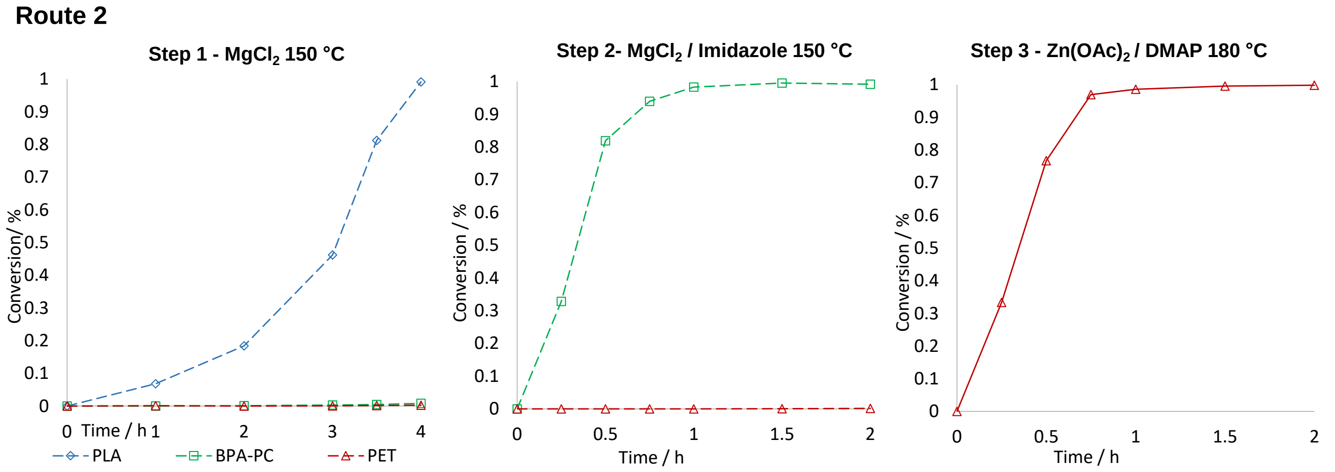

Figure S22. Kinetic plots of conversion against time for the selective and sequential depolymerisation for each step of the Route 2 selective depolymerisation.

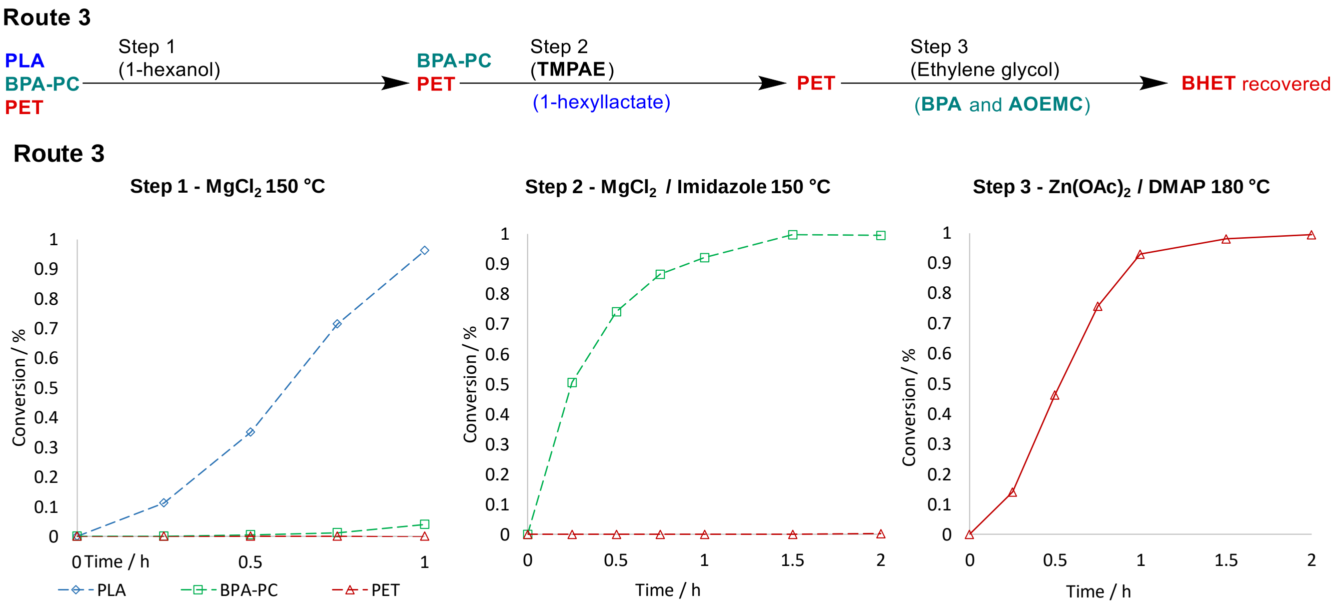

Figure S23. Kinetic plots of conversion against time for the selective and sequential depolymerisation for each step of the Route 3 selective depolymerisation.

References

[1] Petrus, R.; Bykowski, D.; Sobota, P., Solvothermal Alcoholysis Routes for Recycling Polylactide Waste as Lactic Acid Esters. *ACS Catal.* **2016**, *6* (8), 5222−5235.

[2] Sardon, H.; Jehanno, C.; Demarteau, J.; Mantione, D.; Arno, C.; Ruiperez, F.; Hedrick, J.; Dove, A., Selective Chemical Upcycling of Mixed Plastics Guided by a Thermally Stable Organocatalyst. *Angew. Chem. Int. Ed.* **2021**, *60* (12), 6710–6717.

[3] Delle Chiaie, K. R.; McMahon, F. R.; Williams, E. J.; Price, M. J.; Dove, A. P., Dual-catalytic depolymerization of polyethylene terephthalate (PET). *Polym. Chem.*, **2020**, *11* (8), 1450−1453.
